# Protein Kinase C Life Cycle: Explained Through Systems Biology Approach

**DOI:** 10.3389/fphys.2022.818688

**Published:** 2022-04-14

**Authors:** Naveed Aslam, Farah Alvi

**Affiliations:** ^1^ BioSystOmics, Houston, TX, United States; ^2^ Department of Physics, COMSATS University Islamabad, Lahore Campus, Lahore, Pakistan

**Keywords:** PKC, life cycle, activation, down-regulation, lipid

## Abstract

Protein kinase C (PKC) enzymes are a family of kinases that mediate signal transduction originating at the cell surface. Most cell membranes can contain functional PKC enzymes. Aberrations in the PKC life cycle may result in cellular damage and dysfunction. For example, some cancerous cells exhibit alterations in PKC activity. Here, we use a systems biology approach to describe a molecular model of the PKC life cycle. Understanding the PKC life cycle is necessary to identify new drug targets. The PKC life cycle is composed of three key regulatory processes: maturation, activation, and termination. These processes precisely control PKC enzyme levels. This model describes the fate of PKC during *de novo* synthesis and PKC’s lipid-mediated activation cycle. We utilize a systems biology approach to show the PKC life cycle is controlled by multiple phosphorylation and dephosphorylation events. PKC processing events can be divided into two types: maturation via processing of newly synthesized enzyme and secondary messenger-dependent activation of dormant, but catalytically competent enzyme. Newly synthesized PKC enzyme is constitutively processed through three ordered phosphorylations and stored in the cytosol as a stable, signaling-competent inactive and autoinhibited molecule. Upon extracellular stimulation, diacylglycerol (DAG) and calcium ion (Ca^2+^) generated at the membrane bind PKC. PKC then undergoes cytosol-to-membrane translocation and subsequent activation. Our model shows that, once activated, PKC is prone to dephosphorylation and subsequent degradation. This model also describes the role of HSP70 in stabilization and re-phosphorylation of dephosphorylated PKC, replenishing the PKC pool. Our model shows how the PKC pool responds to different intensities of extracellular stimuli? We show that blocking PHLPP dephosphorylation replenishes the PKC pool in a dose-dependent manner. This model provides a comprehensive understanding of PKC life cycle regulation.

## Introduction

Cell membranes are platforms for the transduction of vital signals affecting cellular fate (Newton, 1997; Gould and Newton, 2008; Newton, 2010). Signal transduction must occur for cells to function normally (Gould and Newton, 2008; Newton, 2010). Protein kinase C (PKC) enzymes comprise a family of enzymes that transduce signals originating from the cell surface (Newton, 2010). Signaling involving this family is initiated by receptor-mediated hydrolysis of membrane phospholipids (Newton, 1995a; Keranen et al., 1995; Newton, 1997; Dutil et al., 1998; Gould and Newton, 2008; Newton, 2010). The members of this enzyme family are regulated at the level of structure, function, and subcellular localization (Keranen et al., 1995; Newton, 1995b; Newton, 1996; Newton, 1997; Dutil et al., 1998; Behn-Krappa and Newton 1999; Violin et al., 2003; Gould and Newton, 2008; Newton, 2010). Dysfunction of these enzymes may relate to many human disease pathologies, including cancer, diabetes, and heart disease (Sledge et al., 2006; Palaniyandi et al., 2009; Urtreger et al., 2012; Wallace et al., 2014; Antal et al., 2015; Dowling et al., 2016; Callender and Newton 2017; Isakov, 2018. PKC modulates aspects of cellular physiology, such as angiogenesis, cellular motility, proliferation, differentiation, and apoptosis (Newton, 2003). Differential expression and activity of enzymes in this group has been linked to most types of cancer, including carcinomas (e.g., breast, colorectal), sarcomas (e.g., glioma), lymphomas (e.g., diffuse large B-cell lymphoma) and leukemias (Sledge et al., 2006; Urtreger et al., 2012; Wallace et al., 2014; Antal et al., 2015; Dowling et al., 2016; Isakov, 2018; Ali et al., 2009; Gökmen-Polar et al., 2010). Generally, loss of function is linked to cancer, whereas enhanced activity may relate to neurodegeneration (Callender and Newton 2017). Many groundbreaking investigations during the last two decades have suggested that these enzymes have novel therapeutic potential as drug targets in different cancers, heart failure and neurodegenerative diseases (Keranen and Newton 1997; Newton, 2003; Sledge et al., 2006; Palaniyandi et al., 2009; Urtreger et al., 2012; Wallace et al., 2014; Antal et al., 2015; Dowling et al., 2016; Callender and Newton 2017; Isakov, 2018). Diverse catalytic and regulatory mechanisms exist within the members of this family, providing a plethora of possibilities for drug target design (Newton, 1995a; Edwards and Newton 1997; Keranen and Newton 1997; Mosior and Newton 1998; Nalefski and Newton 2001; Newton, 2003; Newton, 2004; Steinberg, 2008; Antal et al., 2014).

Cellular function depends on the ability of cells to mount dynamic responses to environmental cues. A critical part of this cellular response is the tight modulation of signaling events (Newton, 2009). Phosphorylation is a crucial mechanism to transduce environmental signals from the cell membrane to the cytoplasm. Cellular phosphorylation of substrates is controlled by hundreds of kinases and phosphatases. Global cellular functions (i.e., proliferation and apoptosis) and specialized functions (i.e., hormone secretion) maintain homeostasis through phosphorylation and dephosphorylation of signaling substrates. Imbalances in the phosphorylation states of important substrates may result in a disease state, such as cancer or neurodegeneration. Second messengers like Ca^2+^/DAG transmit crucial signaling information that controls cellular fate. Members of this kinase family bind to secondary messengers, which may play a critical role in translating environmental cues into useful and vital intracellular information by determining the phosphorylation status of key downstream substrates. Intriguingly, the phosphorylation events within the members of this family are constitutive whereas the dephosphorylation processes are agonist-driven (Mosior and Newton 1998; Steinberg, 2008; Antal et al., 2014). Novel therapies may target PKC phosphorylation states.

As the dysfunction of these family members may result in disease states, it is crucial to understand the underlying molecular mechanisms that control cellular levels of PKC. Understanding downstream signaling of these enzymes will aid in rational design of new therapeutic strategies. Here, we propose a molecular model of the PKC life cycle based on a systems biology approach. Our model is based on two key observations: 1) phosphorylation controls PKC enzyme stability; and 2) PKC activation and termination are agonist-modulated events (Mosior and Newton 1998; Steinberg, 2008; Antal et al., 2014). According to this model, since PKC is constitutively phosphorylated, PKC dephosphorylation modulates PKC levels and the amplitude of PKC signaling in cells.

The protein conformation of these family members is under tight control. Conformational control is necessary for PKC to function in a multitude of cell types. Two key molecular events may modulate PKC conformation: 1) events of constitutive phosphorylation; and 2) secondary messenger-dependent membrane binding/unbinding events. Newly synthesized PKC is unstable and quickly undergoes degradation when not modified by phosphorylation events. Naïve PKC undergoes a series of ordered phosphorylation events to avoid degradation events (Newton, 1995b; Edwards and Newton 1997; Keranen and Newton 1997; Newton, 2003; Steinberg, 2008; Zhang et al., 1994). These phosphorylation events stabilize PKC, rendering it a catalytically competent and auto-inhibited enzyme (Newton, 1995a; Steinberg, 2008). Then the catalytically primed, auto-inhibited enzyme is directed for storage at various localities within cells. Localization of PKC is maintained by tethering to multimolecular complexes or to other structures (Newton, 1995b; Steinberg, 2008). Constitutive phosphorylation of PKC serves two main regulatory functions in the life cycle of both conventional and novel PKCs: 1) stabilization of nascent enzyme to prevent degradation; and 2) priming PKC to rapidly activate in response to interaction with secondary messengers (Newton, 1995a; Steinberg, 2008). Stabilization of nascent PKC is crucial because the amplitude and duration of signaling downstream of PKC can be linked to the changes in PKC levels. Nascent PKC stabilization may also affect agonist-induced PKC signaling in cells.

This study proposes a comprehensive model of the PKC life cycle from biosynthesis to degradation. Rationale for the model proposed in this study was derived from many of the following experimental observations: 1) Depleted PKC has been observed in cells lacking kinases, such as PDK1 (Dutil et al., 1998; Balendran et al., 2000). 2) In addition, nascent PKC phosphorylation at activation site is constitutive (Newton, 1995b; Keranen et al., 1995; Newton, 1997; Gould and Newton, 2008; Newton, 2010). 3) It has been shown that PKC must bind to complexes like mTORC_2_ for phosphorylation to occur at the hydrophobic motifs of PKC (Ikenoue et al., 2008). 4) It is known that constitutive phosphorylation regulates PKC stability and catalytic competence (Keranen et al., 1995; Bornancin and Parker 1996; Behn-Krappa and Newton 1999; Edwards et al., 1999; Parker and Parkinson 2001; Newton, 2010). 5) Sustained elevation of the endogenous activator DAG/PMA leads to activation, dephosphorylation and down-regulation of PKC (Newton, 1995a; Edwards and Newton 1997; Keranen and Newton 1997; Newton, 2003; Newton, 2004; Steinberg, 2008; Newton, 2010; Antal and Newton, 2013). 6) Inhibition of PKC active sites protects enzyme from dephosphorylation and results in PKC accumulation in its mature form (Gould et al., 2001). 7) PH domain and Leucine rich repeat Protein Phosphatase (PHLPP)-mediated dephosphorylation controls PKC degradation (Gao et al., 2008). 8) In the absence of chronic second-messenger cell stimulation, PKC isozyme has a long half-life on the order of days (Hansra et al., 1999; Newton, 2010). 9) Finally, a family of 70 kDa heat shock proteins called HSP70 proteins are required for the re-phosphorylation of PKC following PKC activation. Re-phosphorylation protects active PKC from degradation, replenishing the cellular pool of PKC for the next cycle (Gao and Newton 2002; Whitesell and Lindquist 2005; Gao and Newton 2006; Abrahamsen et al., 2012).

Although the basic life cycle model described here reflects one pathway of PKC life cycle, studies indicate that basic model can be extended by incorporating additional complexities. In certain PKC signaling cases the fully phosphorylated form of PKC can also be directly targeted for degradation (Bornancin and Parker, 1996; Parker and Parkinson 2001; Srivastava et al., 2002). In that case there might be two competing pathways of PKC down-regulation, one based on dephosphorylated form and other based on fully phosphorylated form. Incorporating, this additional pathway of degradation into the base model will enhance the agonist-induced down-regulation profiles of the enzyme. The basic model only provides a general activation/down-regulation mechanism for the entire family. It is very much possible that when base model is used to study the activation/down-regulation of individual isoforms additional details such as specific cofactors or binding partners probably also need to be appended in the basic structure to model that specific family member. Interestingly, activation by different agonists can have different effects on the PKC down-regulation and sustained activation may not lead to complete down-regulation (Parker and Parkinson 2001; Lum et al., 2016), this in part we show in our results by perturbing the base model with different intensities and duration of activation stimulation. So, in this case low intensity stimulation may represent agonists such as agonist DAG which may not lead to significant down-regulation whereas, high intensity stimulation such as agonist PMA could lead to significant down-regulation. Another interesting extended model could be mapping the PKC down-regulation in the absence of PHLPP1 (Baffi et al., 2019). In the basic version of life cycle model, HSP70 only provide a protective function by aiding the recovery of enzyme from degradation pathways. Interestingly, some previous observations show that HSP70 may have multiple functions in PKC life signaling (Gao and Newton 2006; Gould et al., 2009; Lum et al., 2013). At least in one case (Lum et al., 2013) HSP70 seems to be stimulating the degradation whereas HSP90 appears to provide the protective function. The basic version does not address the complex roles of individual HSP family members on PKC lifetime and signaling.

In this study, we examine the mechanisms by which the cellular PKC pool is established and maintained through constitutive phosphorylation events after *de-novo* synthesis. In addition, we explore possible molecular mechanisms responsible for PKC activation and down-regulation during agonist-induced stimulation. Understanding the PKC life cycle is crucial to designing therapeutics targeting PKC enzymes to alleviate many human diseases. PKC dysfunction has been correlated with a multitude of disease states. For example, PKCβ protein levels are 4-5-fold higher in breast cancer cells than in normal mammary cells (Li and Weinstein, 2006). Here, we use a systems biology model of the PKC life cycle to show that PKC down-regulation is dependent on the amplitude and the duration of second messenger stimulation. Our results also suggest that PKC down-regulation depends on dephosphorylation pathways. Specifically, on exposure to a larger second messenger pulse stimulation, PKC becomes locked in a dephosphorylated, but active, state. In response to this kind of stimulation, all the cellular PKC pool quickly undergoes degradation. Degradation occurs because the HSP70-mediated re-phosphorylation process slows under these conditions, and can therefore only minorly contribute to rescue, active and dephosphorylated PKC. Finally, our results demonstrate that blocking the PHLPP-mediated dephosphorylation pathways eliminates agonist-mediated PKC down-regulation, even during highly stimulated cellular conditions.

Our findings indicate that the PKC life cycle is subject to tight temporal modulation that occurs through a complex sequence of phosphorylation events. These molecular events are broadly classified as either constitutive or agonist induced. Constitutive events are dominant during the early part of the PKC life cycle, when PKC is most unstable. Agonist-induced events are dominant later in the PKC life cycle, during the activation and down-regulation phases. Over the course of the PKC life cycle, naïve PKC transforms itself into a specific transducer of extracellular information precisely relaying it to a multitude of intracellular signals. Using a systems-based approach, we show that the amount of activated PKC affects cellular PKC levels. The relationship between activated PKC levels and the PKC cellular pool may have significant implications for human disease. Our findings also show that heat shock proteins play a significant role in the re-phosphorylation and re-stabilization of PKC, even during highly stimulated cellular conditions. Our results indicate that even when cells are highly stimulated, PKC can be rescued from degradation. The amount of re-phosphorylated PKC increases in a dose-dependent manner in response to increasing HSP70 protein expression. The results presented in this study provide a kinetic-mechanistic model for the PKC life cycle. This model may be instrumental in drug design targeting members of the PKC enzyme family.

## Results

### A Model of the PKC Life Cycle

**FIGURE 1:**
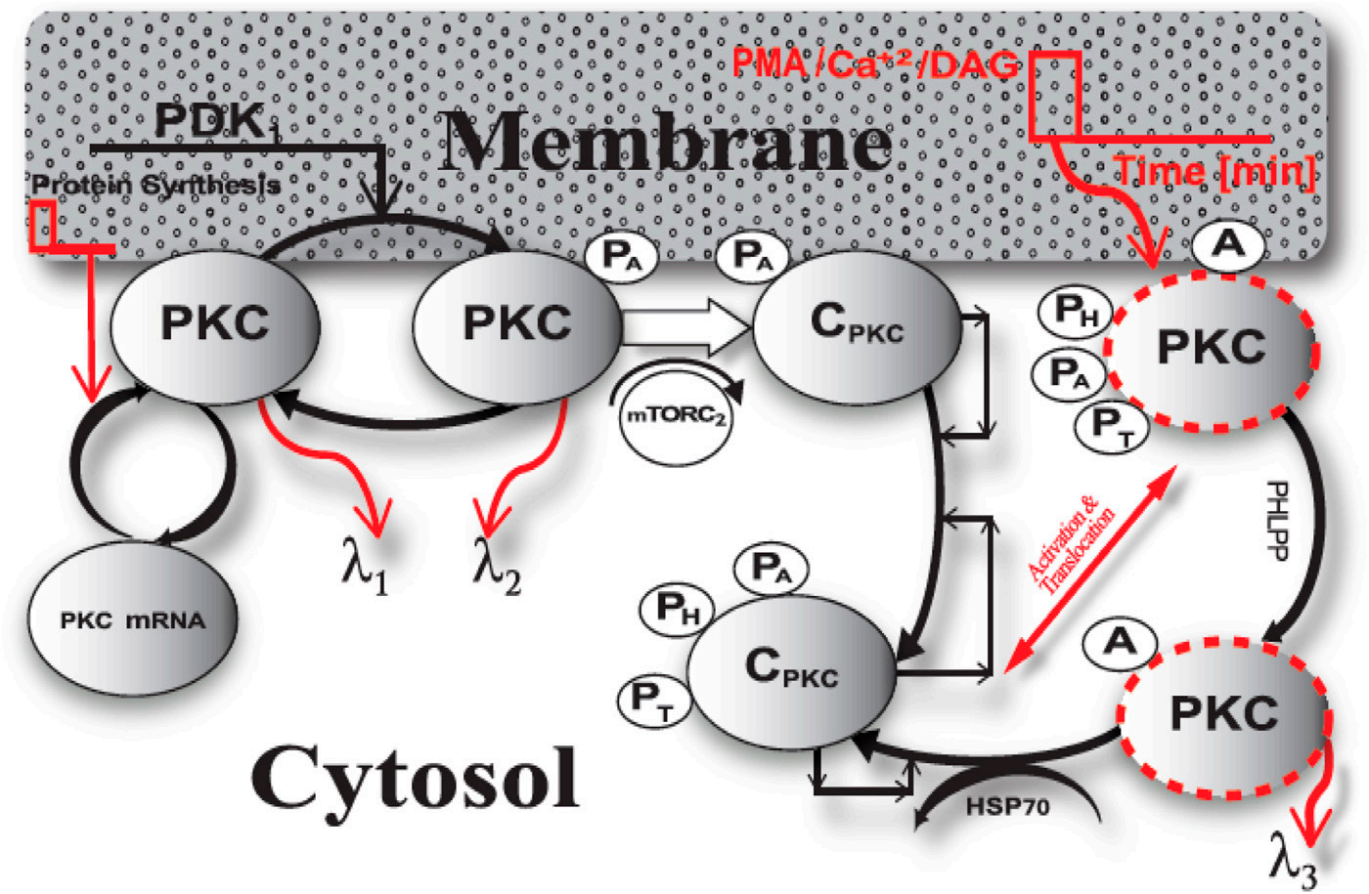
The life cycle model of PKC enzyme. This model explains the fate of naïve, PKC molecule. PKC life cycle has two phases a) constitutive phase and b) agonist-evoked phase. The constitutive phase engages in stabilization and maturation processing of newly synthesized enzyme. Here, the newly synthesized unphosphorylated cPKC is highly unstable. This model describes that constitutively active upstream kinase, PDK1, modulates the phosphorylation of activation loop (P_A_). This initial phosphorylation stabilizes the labile enzyme and triggers two sequential phosphorylation events on the C-terminal tail i.e., hydrophobic and turn motif phosphorylation events (P_H_ & P_T_). These three constitutive ordered phosphorylation events generate a mature, phosphatase/protease resistant molecule (CPKC.P_A_.P_H_.P_T_). As per this model a brief second messenger pulse at the membrane compartment can lead to translocation and activation of mature PKC enzyme and second messenger binding at membrane transforms the inactive mature molecule to active specie i.e., C.PKC.P_A_.P_H_.P_T_^A^. This active molecule is prone to dephosphorylation and on PHLPP-mediated dephosphorylation event relocates back to cytosol where either it can be either degraded or re-phosphorylated through hsp70 and autophosphorylation-mediated events. C.PKC.P_A_.P_H_.P_T_ is cytosolic and on activation stimulation it translocates to membrane. The active form of enzyme at membrane PKC. P_A_.P_H_.P_T_^A^ undergoes PHLPP mediated dephosphorylation and active but dephosphorylated form at membrane i.e., PKC^A^ translocate back to cytosol where it can undergo degradation or re-phosphorylated to become part of a stable pool of the enzyme.

In this study, we propose a model for the PKC enzyme life cycle. This model explains the fate of the nascent PKC molecule. We propose (Figure 1) that temporal regulation of conventional and novel PKCs is based on two phases: 1. constitutive phase; 2. agonist-induced activation/down-regulation/termination phase. In this model, PKC could be either an mRNA (PKC mRNA) or a protein (PKC). The PKC protein can be in one of six states: nascent enzyme at membrane (PKC), enzyme phosphorylated at activation site through PDK1-mediated molecular event at membrane (PKC.P_A_), the complex of PKC. P_A_ with mTORC_2_ at the membrane (C.PKC.P_A_), the enzyme-complex phosphorylated at activation, hydrophobic and turn motifs and located in the cytosol (C.PKC.P_A_.P_H_.P_T_); the activated and phosphorylated enzyme at the membrane (PKC.P_A_.P_H_.P_T_)^A^; and the dephosphorylated but active molecule located in the cytosol (PKC^A^). Here, C.PKC.P_A_.P_H_.P_T_ is cytosolic and on activation stimulation translocates to membrane. The active form of enzyme at membrane PKC.P_A_.P_H_.P_T_^A^ undergoes PHLPP mediated dephosphorylation and active but dephosphorylated form at membrane i.e., PKC^A^ translocate back to cytosol where it can undergo degradation or re-phosphorylated to become part of a stable pool of the enzyme. According to this model, brief protein synthesis stimulation leads to generation of naïve PKC. Newly synthesized, unphosphorylated PKC is highly unstable and only loosely tethered at the cell membrane. Naive PKC is an exposed pseudo-substrate with an accessible C-terminal tail. According to the model, constitutively active PDK_1_ docks with the C-terminal of nascent PKC and modulates PKC phosphorylation at activation loop (P_A_). Constitutive phosphorylation stabilizes the previously labile naïve enzyme and triggers binding to the mTORC2 complex at the cellular membrane. This binding event, in turn, catalyzes a series of sequential autophosphorylations on PKC’s C-terminal tail, hydrophobic motif and turn motif. These phosphorylation events are P_H_ and P_T_. These three constitutive ordered phosphorylation events generate a mature, phosphatase/protease resistant, but catalytically competent molecule denoted as C.PKC.P_A_.P_H_.P_T_. Subsequently, the directions are provided to catalytically competent molecule for cytosolic localization and extended storage. The C.PKC.P_A_.P_H_.P_T_ form of PKC is stable and inactive, but catalytically competent and functionally responsive to second messenger changes inside the cell or at the membrane compartment. According to this model, the first phase of the PKC life cycle involves only constitutive phosphorylation events. During this phase, after a brief protein synthesis pulse, the naïve molecule undergoes transformation into a stable and inactive specie but is still responsive to elevation in the levels of second messenger at the specific membrane locations. In the first phase of the life cycle, PKC is stored at many discrete locations throughout the cytosol. Our model emphasizes how PKC functionality and signaling depends on constitutive processing events, leading to its stabilization and catalytic competence?

This model suggests that, during the second phase of the life cycle, inactive, phosphatase/proteasome-resistant PKC species undergo activation either through second messengers Ca^2+^ and DAG or phorbol myristate acetate (PMA). A brief stimulation pulse at the membrane either generates second messengers Ca^2+^/DAG or PMA. The model is based on prevailing evidence that enzyme reversibly activated by Ca^2+^/DAG binding but irreversibly activated by PMA binding. Thus, the model states that usually Ca^2+^/DAG binding is not strong enough to promote degradation however, PMA binding can lead to significant degradation and down-regulation. Thus, this model states that low intensity pulse stimulation represents Ca^2+^/DAG binding which leads to reversible activation and on the removal of Ca^2+^/DAG, the enzyme is released from membrane and adopts the stable, autoinhibited conformation in the cytosol whereas, the high intensity pulse stimulation represents phorbol esters binding leading to irreversible activation and degradation. Thus, the lower intensity activation pulse, in turn, leads to translocation, reversible activation, and re-translocation to cytosol while higher intensity activation pulse leads to translocation, irreversible activation, dephosphorylation and either, degradation in cytosol or restoration back to dormant form. The model shows that a brief pulse of DAG or PMA in the membrane compartment leads to the translocation of mature PKC from the cytosol to the membrane. At the membrane, binding with DAG/PMA converts inactive PKC to active, but phosphatase-sensitive PKC. This form of PKC is represented by PKC.P_A_.P_H_.P_T_^A^. In this stage, PKC is active but prone to dephosphorylation. PHLPP-mediated dephosphorylation then relocates PKC back to the cytosol. Active but dephosphorylated PKC^A^ is also a target either for degradation or re-phosphorylation by HSP70-mediated rescue and autophosphorylations. Our model suggests that chronic activation of PKC may result in complete dephosphorylation and down-regulation of PKC. PKC’s life cycle involves *de-novo* synthesis, maturation, activation and inactivation/re-migration or termination. Each of these events affects PKC levels and, thus, the agonist-mediated amplitude and duration of active PKC signaling.

The rate parameters of biochemical interactions (**Materials & Methods: R1 -R12**) describing the basic life cycle model are obtained through a systematic strategy described here: 1) following rate constants were obtained from literature: 1) half-time of PDK1-mediated phosphorylation event of nascent, endogenous PKCα enzyme in COS-7 cells at activation loop (Sonnenburg et al., 2001); 2) degradation rate of naïve newly synthesized enzyme (Bornancin and Parker 1996); 3) degradation rate of fully phosphorylated and catalytically competent enzyme in cytosol (Keranen et al., 1995; Newton, 1995a; Newton, 1995b; Bornancin and Parker 1996; Newton, 1996; Edwards and Newton 1997; Keranen and Newton 1997; Newton, 1997; Dutil et al., 1998; Behn-Krappa and Newton 1999; Sonnenburg et al., 2001; Newton, 2003; Violin et al., 2003; Newton, 2004; Gould and Newton, 2008; Steinberg, 2008; Newton, 2010; Antal and Newton 2013; 4) half-time of autophosphorylations (Sonnenburg et al., 2001); 6) the degradation rate of dephosphorylated active enzyme (Lee et al., 1996; Lu et al., 1998; Leontieva and Black, 2004); 7) The PMA mediated down-regulation time (Gould et al., 2011); 8) half-time of PKC maturation (10–30 min for endogenous PKCα and over expressed PKCβII (Gould et al., 2011; Sonnenburg et al., 2001); 2) The other unknown parameters were obtained by hit and trial methodology in which certain numerical values were assigned to these parameters and numerous simulations were developed to match the temporal dynamics of observations described above. Final selection based on best set of parameters is presented in the (Supplementary Materials 2 Table S1). One may wonder how realistic and physiologically relevant this approach for estimation of unknown parameters is and how changes in their numerical values could influence the model output. To address these concerns, we carried out a rigorous sensitivity analysis (Supplementary Materials 3 Table S2; Supplementary Materials 4 Figures S_1_–S_18_) of all the model parameters on model output. This provides the insights to identify which parameters could significantly alter the model results as described in detail in the discussion section.

### PKC Life Cycle: Role of Protein Synthesis and Second Messenger Modulated Activation

**FIGURE 2:**
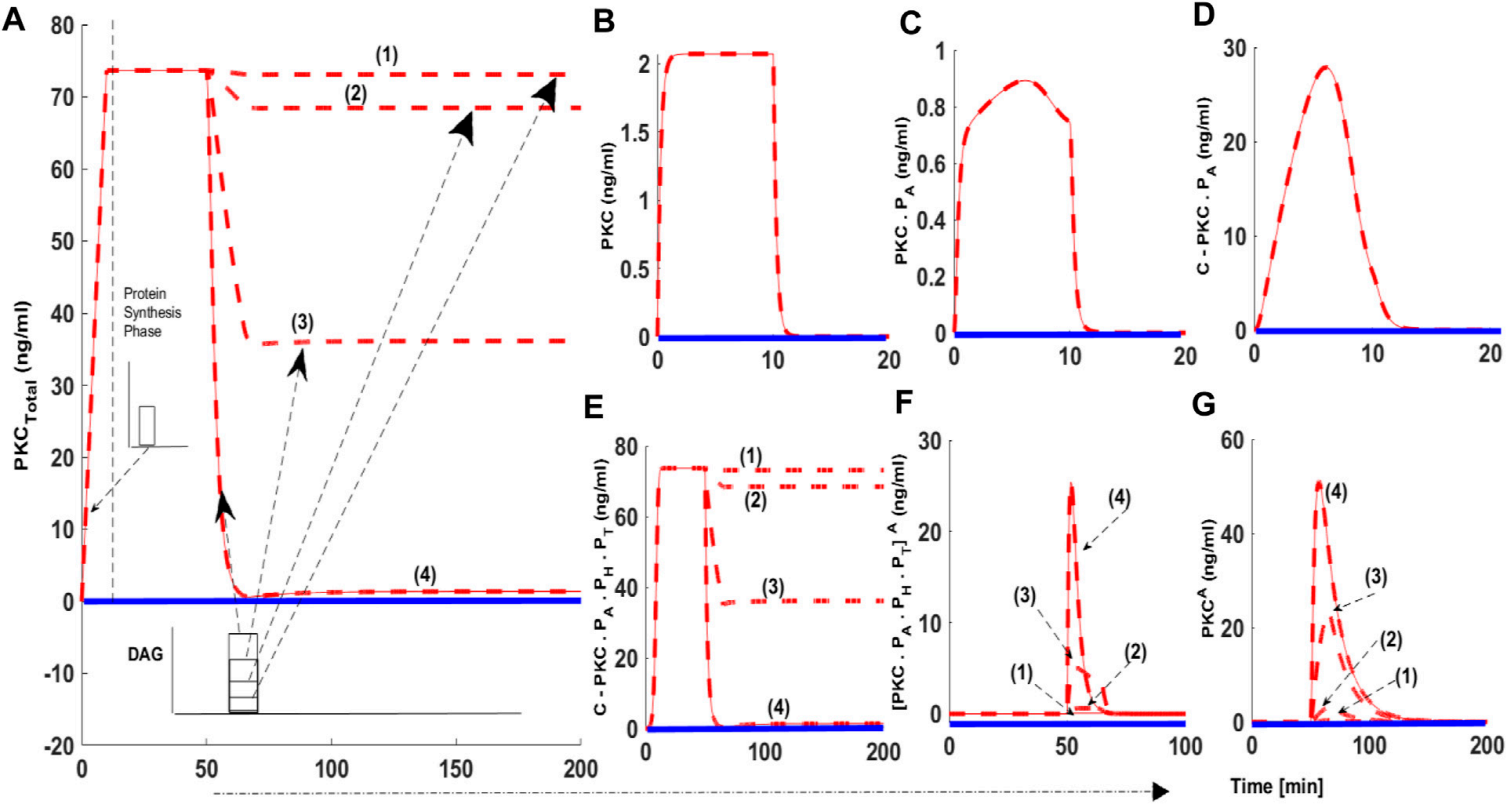
The numerical simulations illustrating the role of protein synthesis and second messenger-mediated activation on cPKC life cycle. These results indicate that initial application of a 10-min protein synthesis pulse leads to the generation of a naïve, and unstable enzyme which through constitutive phosphorylation events at the activation, turn and hydrophobic sites attains stable state. Here, dashed red lines indicate stimulation (protein synthesis as well as second messenger) and blue solid line indicate non-stimulated conditions. **(A)** Total PKC enzyme during protein synthesis and second messenger mediated stimulation. **(B)** concentration of naïve, newly synthesized unstable form of cPKC. **(C)** concentration of PKC species phosphorylated at activation site. **(D)** concentration of PKC complex C. PKCP_A_. **(E)** concentration of PKC complex, phosphorylated at all three sites i.e., activation, turn and hydrophobic sites. These results show that complex C.PKC.P_A_.P_H_.P_T_ is downregulated by higher stimulation levels of second-messenger DAG. **(F)** concentration of active complex PKC. P_A_.P_H_.P_T_.^A^ after second-messenger binding. Again, these results indicate that activation levels of this complex are dependent on the levels of second-messenger stimulation. **(G)** concentration of dephosphorylated but active PKC^A^ molecule.

Using our model, we determined the PKC signaling characteristics like PKC levels and the duration for which these levels remain elevated compared to basal concentrations during protein synthesis, second messenger-modulated activation, and down-regulation events. Signaling characteristics were determined using the levels of total PKC and the relative distribution of all six enzyme species during different phases of the PKC life cycle. We also measured how long total PKC levels and the levels of different forms of PKC are above the basal levels? Here, dashed red lines indicate stimulated conditions like protein synthesis and second messenger-mediated activation. Blue solid lines indicate conditions where there was no stimulation. In these simulations, protein synthesis is initiated by a 10-min pulse, leading to the generation of naïve, unstable PKC. Second messenger stimulation, or a pulse mimicking DAG generation or PMA, is applied 50 min into each simulation. The second messenger pulse is applied for 15 min. Four different levels of activator intensity are used in the pulse stimulation. The activator intensity is set at 0.0005 (nM) and is linked to red dashed line representing (1); intensity set at 0.005 (nM) and is linked to red dashed line (2); intensity set at 0.05 (nM) and is linked to red dashed line (3) and activator (PMA) intensity set at 0.5 (nM) and is linked to red dashed line (4). These different strengths of second messenger/PMA pulse indicate different levels of PKC activation (Figure 2: dashed red lines, (1), (2), 3) and (4)). In the absence of a protein synthesis pulse, there is no *de-novo* PKC synthesis, and the system is fixed in the basal state (Figure 2: solid blue line). Additionally, in the absence of an activator pulse or low intensity stimulation pulse (DAG), there is very little change in total PKC levels (Figure 2: dashed red lines (1)) and transformations into activation states of [ PKC.P_A_.P_H_.P_T_ ]^A^ and PKC^A^ are negligible.

When a protein synthesis pulse is applied the total amount of PKC quickly increases to 73 ng/ml, then stabilizes (Figure 2A). This result shows that, after *de novo* synthesis, newly synthesized PKC is quickly processed through a constitutive mechanism, i.e., three ordered phosphorylation events (Figures 2B–D). PKC is then stored in the cytosol as a stable enzyme competent for signaling, denoted in our model as C.PKC.P_A_.P_H_.P_T_ (Figure 2E). This first phase of the PKC life cycle lasts between 0–50 min (till the application of second messenger pulse stimulation) and illustrates the role of PDK_1_, mTORC_2_, and autophosphorylations in converting labile PKC to a stable, mature, and inactive but catalytically competent molecule. Stable PKC is stored in the cytosol where it then relays second messenger information from the cell surface to key intracellular targets on the interior of the cell. During this first phase of the PKC life cycle the concentration of both the active species i.e., [PKC.P_A_.P_H_.P_T_]^A^ and PKC^A^ are negligible, as no second messenger stimulation has been applied. Both these species remain at basal level concentration during this phase.

These numerical simulations also illustrate that later point, i.e., 50 min into simulation, when a second messenger DAG pulse is introduced, varying levels of the pulse leads to changes in total PKC levels (Figure 2A dashed red lines (1), (2), (3)). Levels of three states of cPKC isozymes i.e., C.PKC.P_A_.P_H_.P_T_ (Figure 2E), PKC. P_A_.P_H_.P_T_.^A^ (Figure 2F) and PKC^A^ (Figure 2G) also change. These results show that second messenger-modulated stimulation initiates the second phase of cPKC life cycle. This phase could possibly involve translocation, activation, termination, and down-regulation events. These results highlight how cPKC down-regulation depends on the duration and strength of the second messenger pulse? In our simulation, we used four different levels of DAG stimulation. Our results show that at the lowest level of second-messenger stimulation, 0.0005 (nM), there is no down-regulation of cPKC. At the highest level of stimulation, 0.5 (nM) cPKC is completely down-regulated (Figure 2A; dashed red lines (1); (2); (3); and (4)). Interestingly, these results also show that PKC down-regulation depends upon PKC.P_A_.P_H_.P_T_.^A^ and PKC^A^ states (Figures 2F,G). In the case of a strong second-messenger pulse (dashed red line (4)), the concentration of PKC.P_A_.P_H_.P_T_.^A^ and PKC^A^ reach their highest levels, leading to significant degradation of PKC. These results also underscore the importance of balance between PKC re-phosphorylation and degradation in controlling the overall PKC concentration after second messenger stimulation.

**FIGURE 3:**
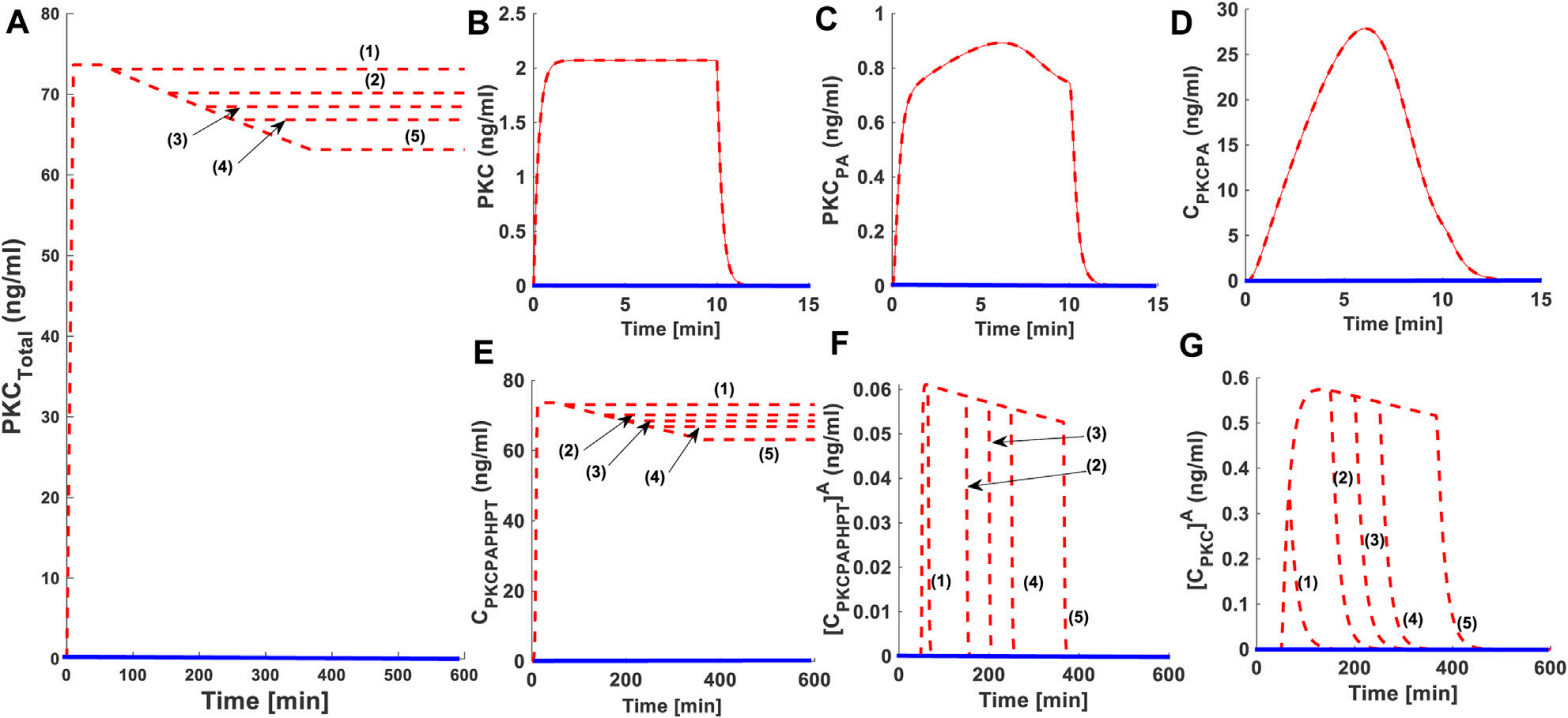
The effect of second messenger-modulated activation duration on the life cycle characteristics of PKC. Here, solid red lines indicate stimulation (protein synthesis as well as second messenger) and blue solid line indicate non-stimulated condition. These results show that during lower intensity second messenger stimulation increasing the duration of pulse can influence the life cycle characteristics of enzyme. However, the degree of this influence is not significant. **(A)** Total PKC enzyme during protein synthesis and second messenger mediated stimulation. These results show that application of a brief pulse protein synthesis pulse (10 min) shows the quick generation of cPKC enzyme and stabilization. These results also show that at later time point (i.e., 50 min) the application of a lower intensity second messenger pulse (15-min pulse mimicking Ca^+2^/DAG generation; DAG = 0.0005 nM) leads to the activation, termination, down-regulation and restoration of cPKC enzyme. These results high-light that cPKC signaling characteristics are only moderately influenced by the duration of second messenger pulse. Here, five different durations of pulse stimulation are used i.e., second messenger DAG pulse stimulation set at 15 min (dashed red line (1)); 100 min (dashed red line (2)), 150 min (dashed red line (3)), 200 min (dashed red line (4)) and 316.66 min (dashed red line (5)) (from shorter to longer duration periods). These results elucidate that at shorter pulse period i.e., 15 min degree of down-regulation is only minimum and at longest pulse period i.e., 316.66 min the degree of down-regulation is only moderate. **(B)** The concentration of naïve, newly synthesized unstable form of cPKC. **(C)** The concentration of PKC species phosphorylated at activation site. **(D)** The concentration of PKC complex C.PKC.P_A_. **(E)** The concentration of PKC complex, phosphorylated at all three sites i.e., activation, turn and hydrophobic sites. **(F)** The concentration of active complex PKC.P_A_.P_H_.P_T_^A^ after second messenger binding. **(G)** Concentration of dephosphorylated but active PKC^A^ molecule.

As part of our investigations, we also analyzed how second messenger pulse duration affects the PKC life cycle. We set DAG pulse intensity to 0.0005 (nM) and varied the duration of this low-level pulse. In these simulations (Figure 3) we used five different durations of pulse stimulation: 15 min dashed red lines (1); 100 min dashed red line (2), 150 min dashed red line (3), 200 min dashed red line (4) and 316.66 min dashed red line (5). Our results show that increasing the duration of the second messenger pulse has only a moderate effect on the PKC life cycle (Figure 3). For lower intensity stimulation, the degree of down-regulation is almost negligible when pulse stimulation is set at 15 min (Figure 3: dashed red line (1)) and is only moderate when pulse stimulation is set at 316.66 min (Figure 3: dashed red line (5)).

### Effect of Blocking PKC Dephosphorylation

**FIGURE 4:**
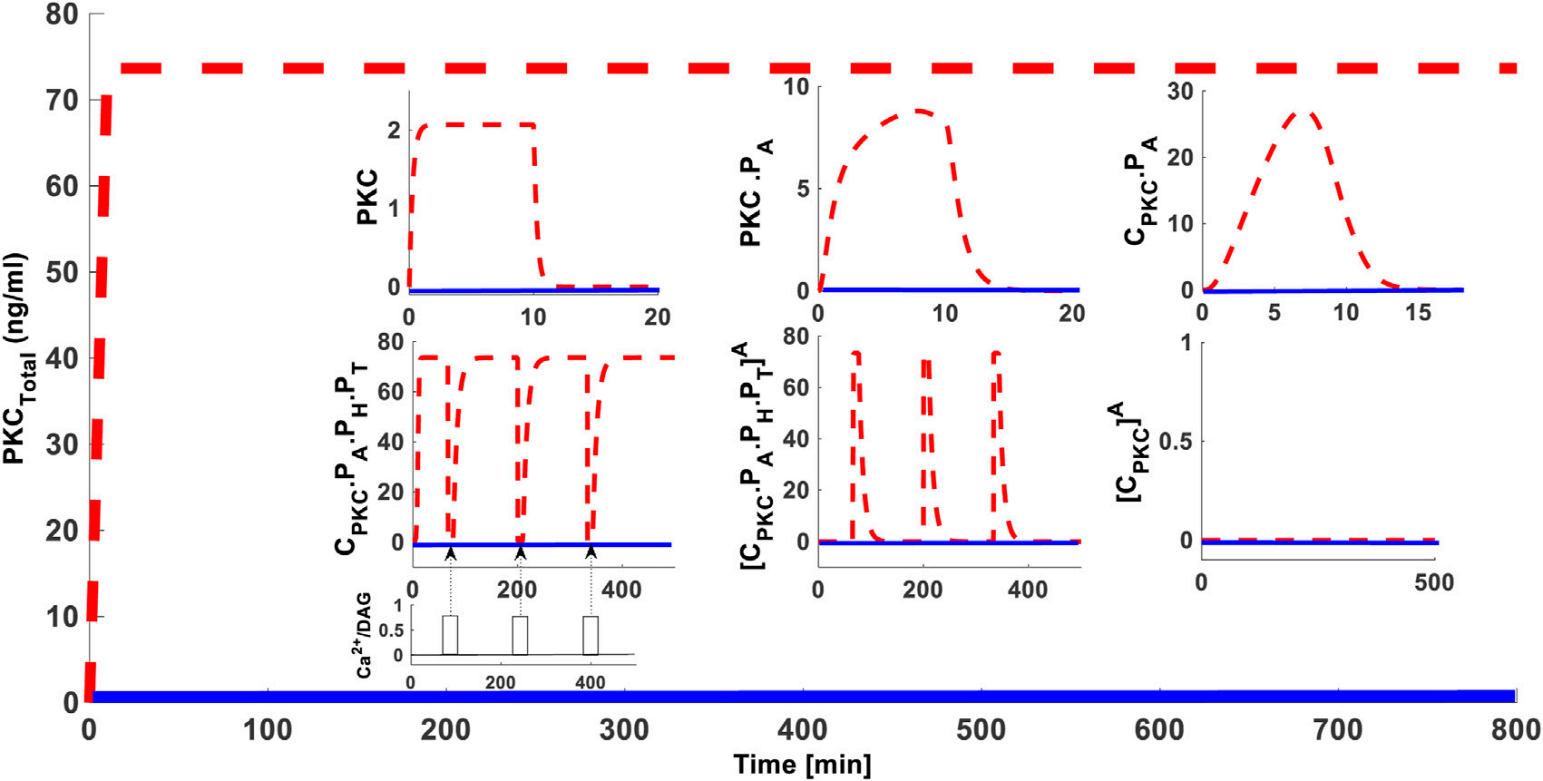
The effect of 100% blocking of dephosphorylation rate i.e., “k_17_”, on the cPKC life cycle. These results show that 100% blocking of dephosphorylation rate prevents the down-regulation of total PKC, even at higher stimulation levels. Here, application of initial protein synthesis stimulation leads to the quick generation and constitutive stabilization of cPKC enzymes. After initial protein synthesis stimulation three identical pulse stimulations of second messenger DAG are applied at time points t = 4,000 s; t = 12,000 s; and t = 20,000 s. Each of these pulse stimulations is applied for 15 min and during their application the dephosphorylation rate k_17_ was completely blocked. These results indicate that blocking the dephosphorylation did not result in the down-regulation of total PKC enzyme as well as the CPKC.P_A_.P_H_.P_T_. The inset shows that application of a second messenger stimulation results in decrease of CPKC.P_A_.P_H_.P_T_ and increase in PKC^A^ but after second messenger is removed the CPKC.P_A_.P_H_.P_T_ levels are restored indicating that down-regulation is very much dependent on the dephosphorylation pathways.

We next determined whether blocking PKC dephosphorylation could influence the PKC life cycle. In these simulations, the protein synthesis protocol is the same as described in the previous section. We applied three sequential pulses of PMA (since its’ high intensity stimulation) during simulations (time = 4,000 s; = 12,000 s; & = 20,000 s). The strength of these sequential pulses was 0.5 (nM) with a fixed duration of 15 min. During these activation pulses dephosphorylation is inhibited by completely blocking the parameter k_17_. Our results show that blocking dephosphorylation prevents down-regulation of cPKC even at higher stimulation levels (Figure 4 dashed red lines for total enzyme and inset for different states of the enzyme). These results show that, if dephosphorylation is blocked, PKC levels are maintained. This shows that high-intensity, activation-induced down-regulation of PKC can take place through dephosphorylation. These results imply that if dephosphorylation could be controlled, PKC down-regulation could be fine-tuned. Interestingly, sequential application of second messenger pulse stimulations can lead to decreases in C.PKC.P_A_.P_H_.P_T_ concentration and increases in PKC.P_A_.P_H_.P_T_^A^ concentration (Figure 4 inset dashed red lines for different states of the enzyme). However, once PMA is removed, C.PKC.P_A_.P_H_.P_T_ and total PKC levels are restored back to their original values. These results emphasize the essential role of different enzyme states in response to second messenger stimulation.

### Effects of sequential intermediate strength second messenger pulse stimulations on the PKC life cycle

**FIGURE 5:**
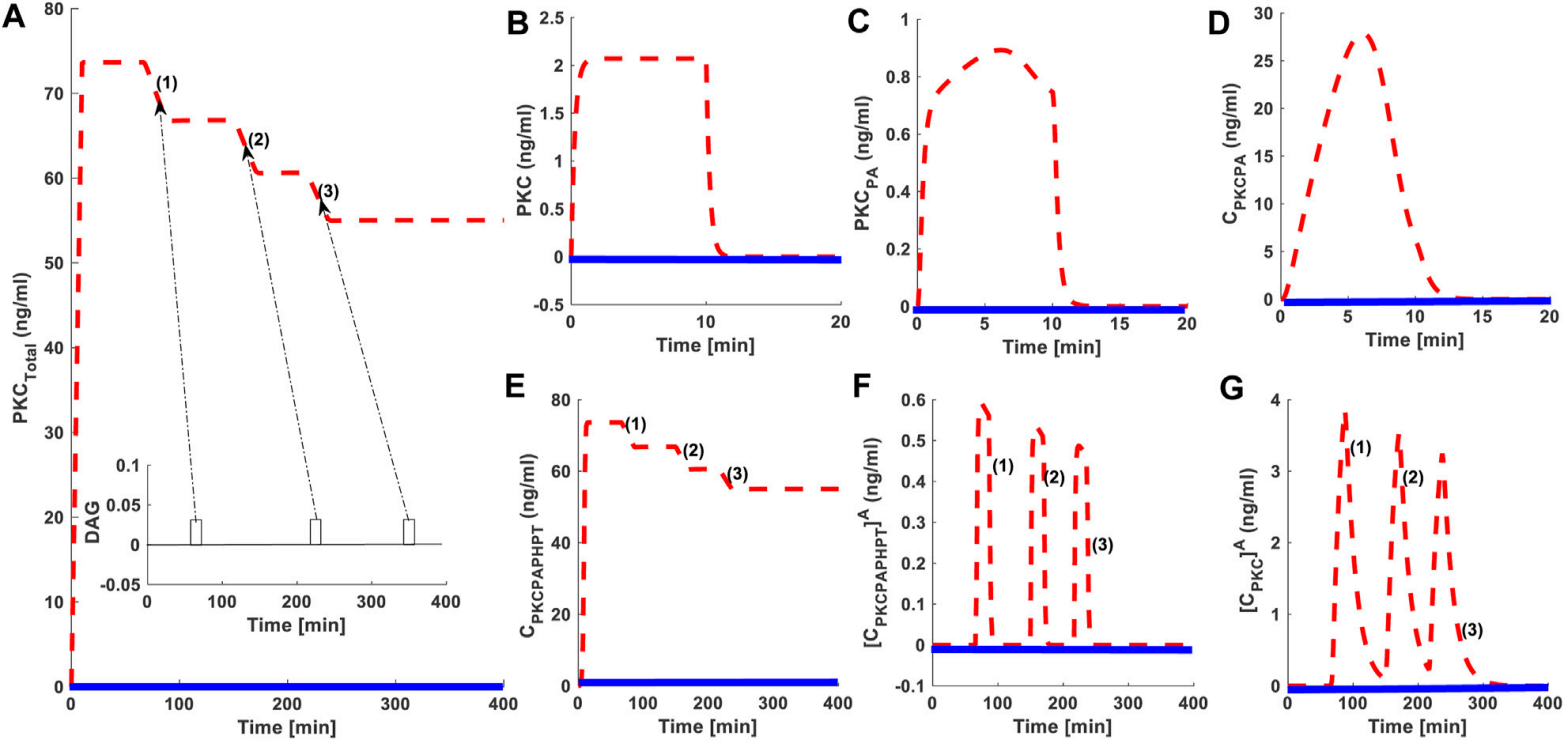
The effect of sequential application of second messenger stimulation on the down-regulation characteristics of the cPKCs. Here, dashed red lines represents the stimulation whereas, the solid blue line represents non-stimulated conditions. Here, three equal pulses of second messenger are applied in a sequential manner. The pulse 1 is applied at t = 50 min; the pulse 2 is applied at t = 220 min; and the pulse 3 is applied at t = 360 min. For all these three pulses second messenger levels are set at DAG = 0.005 nM; These results show the second messenger-induced destabilization and down-regulation of cPKCs. These results also show that with application of each pulse, a certain amount of enzyme is downregulated, and total PKC levels are stabilized at new levels. These results indicate that during simulations three down-regulation events are observed each in response to a second messenger pulse at t = 50 min; t = 220 min; and t = 360 min. **(A)** Total PKC enzyme during protein synthesis and second messenger mediated stimulation. **(B)** The concentration of naïve, newly synthesized unstable form of cPKC. **(C)** The concentration of PKC species phosphorylated at activation site. **(D)** The concentration of PKC complex C.PKC.P_A_. **(E)**The concentration of PKC complex, phosphorylated at all three sites i.e., activation, turn and hydrophobic sites. **(F)** The concentration of active complex PKC. P_A_.P_H_.P_T_^A^ after second messenger binding. **(G)** Concentration of dephosphorylated but active PKC^A^ molecule.

As shown in previous sections, PKC down-regulation can be modulated through varying the strength and duration of second messenger pulse stimulations. Next, we set out to investigate whether sequential intermediate strength second messenger pulses affect the PKC life cycle. Three identical strength second messenger pulse stimulations were applied at three different time points. The first pulse was applied at 50 min; the second pulse was applied at 220 min; and the third pulse was applied at 360 min. For all three pulses, DAG pulse strength is set at an intermediate level (Figure 2A dashed red line (2) and pulse associated with it; DAG = 0.005 nM). Our results indicate that second messenger-induced destabilization and down-regulation takes place when pulse stimulation is applied. PKC down-regulation occurs in response to pulse stimulation, but overall, down-regulation depends on the number of pulses applied (Figure 5A). These results also indicate three down-regulation events occur in response to three pulse stimulations (Figure 5). Interestingly, the extent of down-regulation decreases with each sequential pulse event. After the first pulse, PKC levels drop almost 10 ng/ml. In comparison, for the third pulse, PKC levels drop is only 5 ng/ml. This observation shows that, as the number of pulses increases, the net effect of the pulses on PKC degradation and down-regulation also decreases. This occurs even though the strength of each pulse remains constant. The same phenomenon is true for PKC.P_A_.P_H_.P_T_^A^ and PKC^A^ (Figures 5F,G).

### How HSP70 Proteins May Influence the PKC Life Cycle?

**FIGURE 6:**
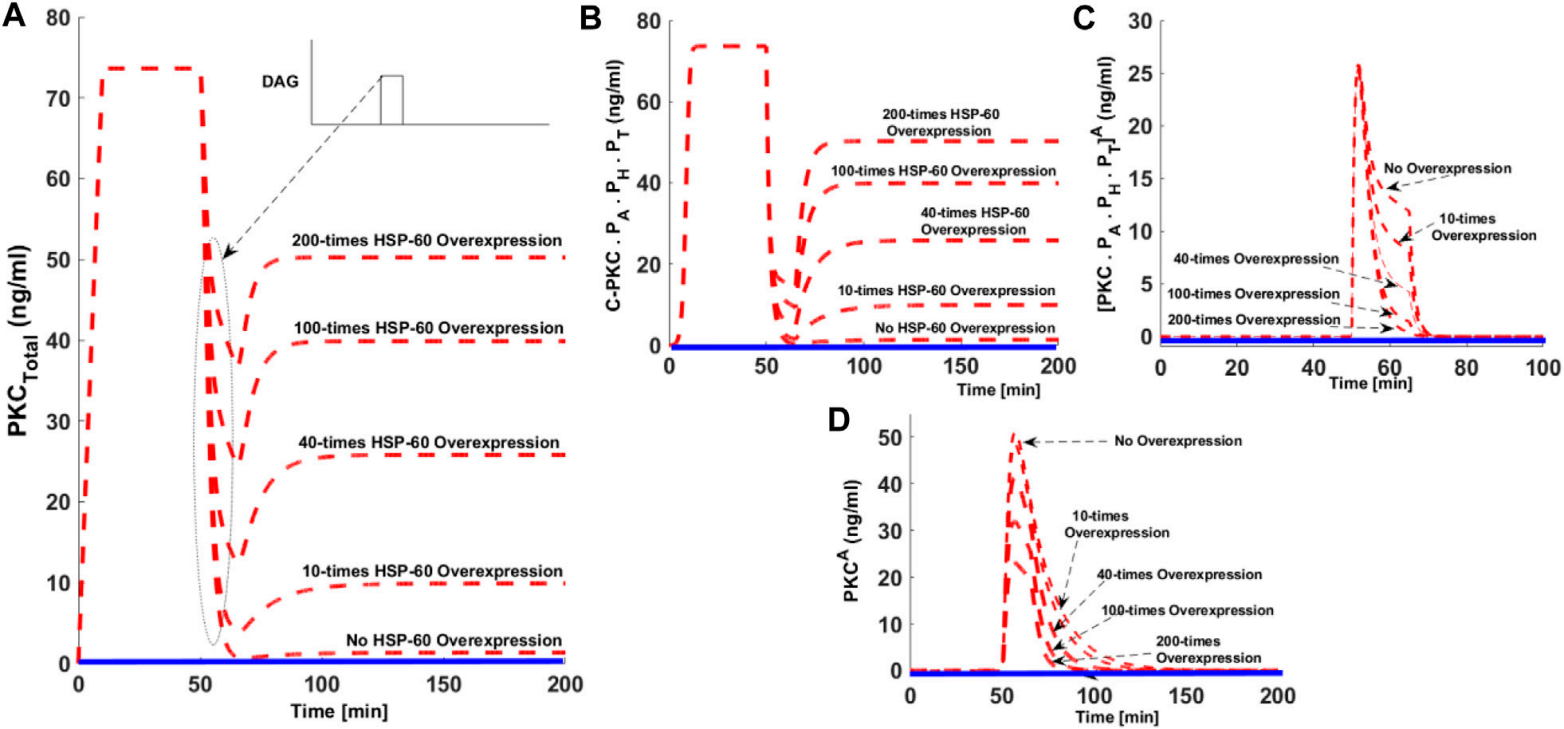
The effect of hsp70 overexpression on the cPKC life cycle. Here, the dashed red line shows stimulated conditions whereas, solid blue line show non-stimulated conditions. **(A)** The concentration of total PKC enzyme. Here, under the no hsp70 overexpression condition the strong second messenger stimulation leads to the complete down-regulation of cPKC enzyme. Interestingly, when these simulations are run with hsp70 overexpression the degree of down-regulation after second messenger stimulation is reduced in a dose-dependent manner. These results indicate the possible role of hsp70 expression in re-stabilization of active but dephosphorylated PKC enzyme. **(B)** The concentration of C-PKCP_A_. P_H_.P_T_ with no hsp70 overexpression and dose-dependent reversal of C.PKC.P_A_.P_H_.P_T_ concentration with hsp70 overexpression. **(C)** The concentration of [PKC.PA.PH.PT]^A^ with no hsp70 overexpression and dose-dependent reduction of [PKC.PA.PH.PT]^A^, concentration with hsp70 overexpression. **(D)** The concentration of PKC^A^, with no overexpression and dose-dependent reduction in concentration and time persistence with hsp70 overexpression.

Previous studies indicate heat shock proteins may prevent agonist-associated dephosphorylation and subsequent down-regulation of cPKC enzymes (Bornancin and Parker 1996). PKC-HSP70 interactions could be important in certain cancers, where overexpression of HSP70 may prevent the loss-of-function (LOF). LOF seems to be associated with the down-regulation of cPKC enzymes. We set out to investigate whether overexpression of heat shock proteins could rescue PKC from degradation. In order, to study this question, we developed five simulations: wild-type with no-HSP overexpression; 10x overexpression; 40x overexpression; 100x overexpression, and 200x overexpression (Figure 6). In these simulations, second messenger pulse strength is set to 0.5 (nM). Here, dashed red lines show the stimulated conditions, whereas the solid blue lines indicate non-stimulation conditions. These results indicate that when there is no HSP70 overexpression, second messenger stimulation can lead to complete down-regulation of PKC (Figures 6A, dashed red line, no HSP70 overexpression). On model perturbation with varying levels of HSP70 overexpression, the degree of cPKC enzyme down-regulation can be reversed in a dose-dependent manner (Figure 6A). These results indicate the possible role of HSP70 expression in PKC rescue. These results show that heat shock protein expression may be instrumental in PKC recovery. C.PKC.P_A_.P_H_.P_T_, also shows complete down-regulation under no HSP70 overexpression and subsequent reversal in a dose-dependent manner on HSP overexpression (Figure 6B). Interestingly, the overexpression of HSP70 reduces the activation and duration of both PKC.P_A_.P_H_.P_T_^A^ and PKC^A^ in a dose-dependent manner, (Figures 6C,D). Heat shock proteins rescue enzymes from degradation and induce re-phosphorylation and autophosphorylation. These results confirm heat shock proteins could be the key to maintaining the cellular pool cPKC after activation. Heat shock proteins can specifically interact with active and dephosphorylated cPKC enzymes, thus reducing cPKC concentration.

### Effects of Blocking PHLPP on the cPKC Life Cycle

**FIGURE 7:**
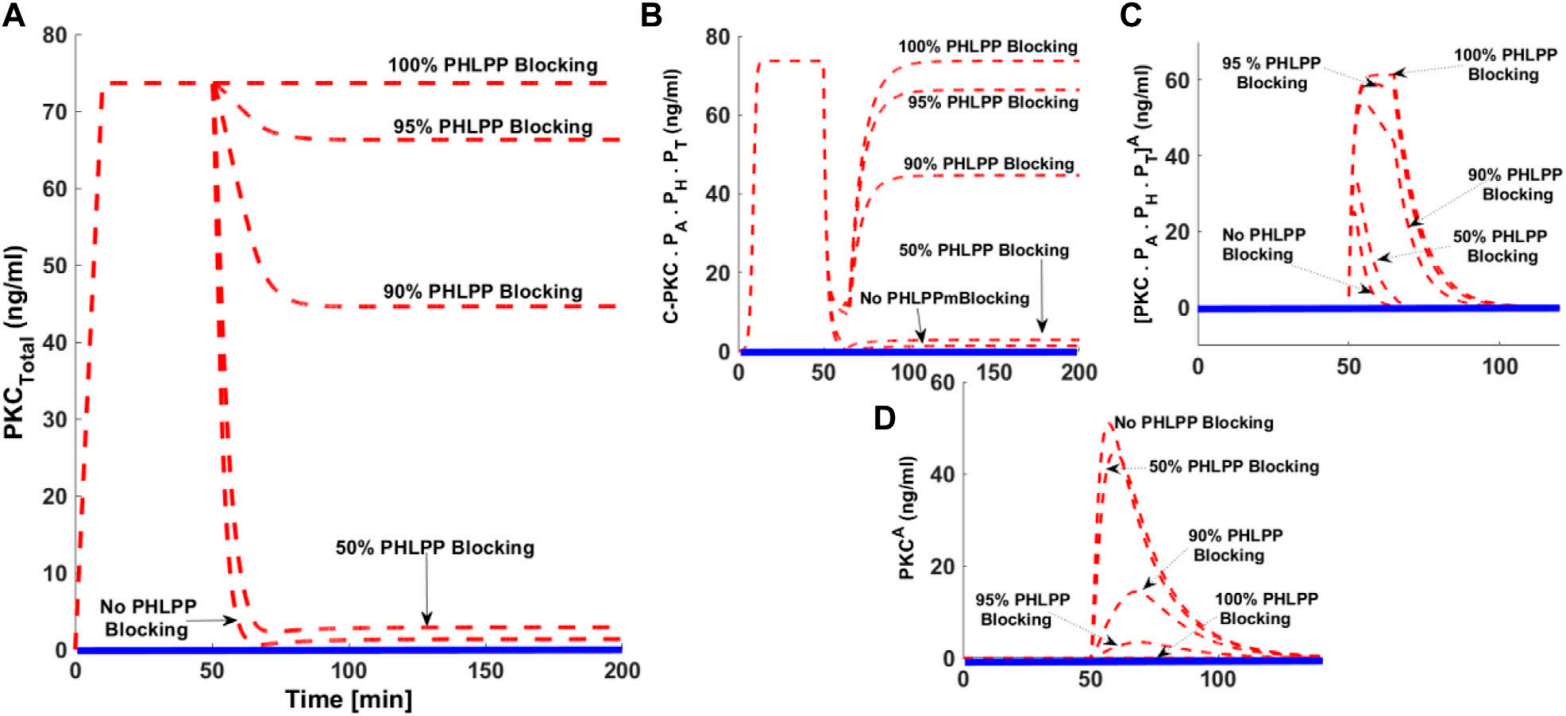
The effect of PHLPP blocking on the down-regulation of cPKC enzyme after second messenger-induced activation. Here, dashed red lines show the stimulation whereas, the solid blue lines show the non-stimulation conditions. **(A)** The concentration of total PKC. These results indicate that after second messenger stimulation (second messenger DAG = 0.5 nM, with pulse duration of 15 min), the cPKC is completely downregulated when there is no blocking of PHLPP. These results show that degree of cPKC destabilization and its down-regulation is reduced in a dose-dependent manner with increasing the blocking levels of PHLPP. These results indicate that with 100% blocking of PHLPP there is no down-regulation of cPKC, even with high strength second messenger pulse stimulation. **(B)** concentration of C.PKC.P_A_.P_H_.P_T_ with no blocking and different blocking levels of PHLPP. **(C)** Concentration of PKCP_A_. P_H_.P_T_.^A^ with no PHLPP blocking and dose-dependent blocking **(D)** Concentration of PKC^A^ with no PHLPP blocking and different levels of PHLPP blocking.

The protein phosphatases PHLPP_1_ and PHLPP_2_ are endogenous negative regulators of cPKC signaling. Both these phosphatases play a key role in regulating the dephosphorylation process of cPKC in different cell types. Studies indicate that both phosphatases can bind and dephosphorylate PKCβII on the hydrophobic motif, thus shunting it towards degradation. These phosphatases have also been linked to cancer. We investigated how blocking PHLPP signaling affected the cPKC life cycle? We addressed this question by developing five simulations mimicking the cPKC life cycle. Our simulations include no PHLPP blocking, 50% blocking, 90% blocking, 95% blocking, and 100% blocking. Here, the dashed red line shows activation upon second messenger pulse stimulation. The solid blue line represents no stimulation. PMA levels were set at 0.5 (nM). With no PHLPP blocking cPKC is completely down-regulated (Figure 7A dashed redline, No PHLPP blocking). This shows cPKC down-regulation can be reduced in a dose-dependent manner culminating when100% PHLPP blocking results in no down-regulation. Even with high intensity stimulation, the complete blocking of PHLPP results in no down-regulation (Figure 7A). C.PKC.P_A_.P_H_.P_T_ levels increase as PHLPP blocking increases. 100% PHLPP blocking increases the concentration and duration of C.PKC.P_A_.P_H_.P_T_^A^. No PHLPP blocking leads to smaller increases in C.PKC.P_A_.P_H_.P_T_^A^ concentration and a shorter duration of persistence (Figure 7C).

## Discussion

Over the past two decades, numerous metabolic and signaling pathways have been linked to enzymes of the PKC family (Mochly-Rosen et al., 1991; Mochly-Rosen and Gordon, 1998; Maruvada and Levine 1999; Murray et al., 1999; Gökmen-Polar et al., 2001; Gao and Newton 2002; Yu et al., 2003; Poole et al., 2004; Larsson, 2006; O'Neill et al., 2011; Kang, 2014). PKC family members are critical for assembly of key transduction complexes. These complexes translate environmental cues into critical physiological processes regulating apoptosis, proliferation, gene expression, cell migration, differentiation, and cell survival. Due to the PKC enzymes’ wide range of influence on physiological processes, members of this enzyme family could serve as significant targets for drug development. They may lead to novel therapies for human diseases such as cancer, heart failure and neurodegenerative conditions. The functional role of cPKCs in cancer is not fully clear. To better understand the complex functional behavior of PKC in cancer, it is important to understand the regulatory processes involved in PKC modulation. Dissecting the molecular underpinnings of relevant regulatory events linked to individual PKC isoforms is key. Understanding the variations of these regulatory processes across the cell types and even between the tumors could be key in establishing safe and effective therapeutic strategies targeting PKC family enzymes.

Our study focused on understanding the PKC life cycle through a systems biology approach. Here, we proposed a computational model based on elementary kinetics to describe distinct phases of the PKC life cycle. The proposed model accounts for the maturation, activation, and termination phases of the enzyme life cycle. We investigated a general hypothesis that levels of PKC enzyme, and hence the duration and amplitude of associated signaling in cells, are under precise control by three key regulatory processes. Any dysfunction in PKC regulation may have a link to disease conditions like cancer. Our proposed life cycle model is based on observations noting that newly synthesized PKC is unstable, and subsequently processed through constitutive ordered phosphorylation events. Activation and termination are agonist-modulated processes and depend on the strength and duration of agonist stimulation.

The proposed network model of PKC life cycle suggests that duration and levels of PKC signaling are regulated by key enzyme interactions during stabilization, activation, and down-regulation phases. According to this model, the stabilization and maturation of newly synthesized molecule is modulated by constitutively active PDK1, mTORC_2_ complex and autophosphorylations. Similarly, the translocation of PKC from the cytosol to the cell membrane and second messenger Ca^2+^/DAG and PMA binding play key roles in activation. The phosphatases PHLPP1 and PHLPP2 control the enzyme down-regulation. Interestingly, hsp70 modulates the re-phosphorylation event, which can rescue PKC from degradation and target it for storage in an auto-inhibited form in the cytosol. Our results suggest that the duration and strength of cPKC signaling in a multitude of cell types can be modulated by PDK1, mTORC2, PHLPP1, and hsp70. Our results are consistent with experimental observations showing the effects of these kinases and phosphatases on PKC signaling during distinct phases of the PKC life cycle.

Our study shows that PKC signaling can be fine-tuned by the duration and amplitude of agonist-induced stimulation (DAG or PMA Figures 2, 3). Our results show that for small to moderate activation stimuli the pool of total PKC remains unchanged, or changes are relatively small (Figure 2A: dashed red line (1) and dashed red line (2); mimicking the DAG based reversible activation). However, high intensity stimuli can lead to complete down-regulation of PKC through activation and dephosphorylation (Figures 2A,E–G: dashed red line (3) and (4); mimicking the PMA based irreversible activation). This occurs because, during the application of higher intensity stimuli, a larger pool of enzyme is activated (Figure 2F: dashed red line (3) and dashed red line (4) mimicking the PMA based irreversible activation). A larger pool of active enzyme results in much higher concentrations of active, but dephosphorylated, enzyme (Figure 2G: dashed red line (3) and dashed red line (4) mimicking the PMA based irreversible activation). These results link the larger pool of active and dephosphorylated PKC to higher degrees of down-regulation. Our model predicts that in the wake of high intensity stimulation, endogenous heat shock protein-induced restoration and recovery of cPKC has only limited influence. When there is a small pool of active and dephosphorylated enzymes, the endogenous heat shock-mediated restoration may completely or partially recover PKC from degradation. However, for much larger pools, the enzyme degrades more quickly compared to its re-phosphorylation and replenishment in the cytosol by endogenous recovery and restoration factors. This model suggests a delicate balance between degradation and re-phosphorylation pathways controls the degree of PKC down-regulation after agonist-mediated activation. One may also wonder that why the activation induced down-regulation of enzyme as described in Figures 2, 3 is taking place on a faster time scale (minutes) whereas observations (Parker and Parkinson 2001; Gao and Newton 2006; Gould et al., 2009; Lum et al., 2013) show that activation induced down-regulation takes place on a slower time scale (2–3 h). This is because in the basic version of model we set the degradation rate of dephosphorylated but active form at the same numerical value as of newly synthesized enzyme. This was done to keep model simple. However, our additional simulations (Supplementary Materials 4 Figure S_19_) show that as the degradation parameter of dephosphorylated form is changed to slower rate the down-regulation after activation appears to take place on the slower time scale. Also, even in the original results of basic model of Figures 2, 3 the realistic representative of activation induced down-regulation is the specie PKC^A^ (Figure 2G: dashed red line (3) and dashed red line (4) mimicking the PMA based irreversible activation) and in these results it shows the down-regulation over the scale of hours. Total PKC (Figure 2A: dashed red line (4) mimicking the PMA based irreversible activation) though goes down rapidly but there is small amount still available due to re-phosphorylation-based recovery which degrades slowly.

Intriguingly, one prediction of our model show that blocking the dephosphorylation event of active form of enzyme at membrane can prevent the PKC down-regulation (Figure 4). One might wonder is there any physiological relevance for this? At least one previous observation in COS7 cells (Gould et al., 2011) show that active site inhibitors can prevent the dephosphorylation and subsequent down-regulation of active PKC. Indirectly, this observation may provide some relevance to our models’ prediction (Figure 4). Though one may criticize this linkage as here we are blocking the dephosphorylation event by rate constant k_17_ in contrast to experiments where massive inhibitor molecules were used (Gould et al., 2011) to mask the active sites which prevents the access of phosphatases and thus down-regulation. So here we assume that blocking rate parameter k_17_ is equivalent to reducing or completely preventing the access of phosphatases to active sites.

Our results also analyze the effects of sequential stimulations and complete blocking of dephosphorylation pathways on PKC activation and down-regulation. Dephosphorylation events are critical for targeting active molecules towards degradation. However, down-regulation after high intensity second messenger stimulation might be completely avoided if dephosphorylation pathways are blocked. Our results show that enzyme down-regulation could be completely avoided when dephosphorylation is blocked even under high intensity, sequential second messenger-mediated activation stimuli (Figure 4: mimicking the PMA based irreversible activation). Our results also predict that, when dephosphorylation pathways are not blocked, the sequential application of high-intensity stimuli can lead to successive enzyme down-regulation events (Figure 5 mimicking the PMA based irreversible activation). However, the degree of down-regulation is reduced with the application of each pulse in a series, despite the strength and duration of the pulses remaining identical (Figures 5A,E–G). This raises an interesting possibility that, initially when PKC levels are high, it might be easier to down-regulate the enzyme. With each cycle the degree of down-regulation could be reduced, raising the possibility that PKC might persist for longer periods after certain initial stimulations in the absence of new protein synthesis or PKC transcription.

In our model, we are not attempting to simulate PKC protein synthesis in a detailed manner, we only intend to mimic the complex signaling cascade involved in new protein synthesis by introducing a 10-min pulse (Figure 2). The pulse stimulation primes PKC-mRNA and stimulates the new synthesis of PKC enzyme. Clearly, a complete model of protein synthesis should account for all the complex molecular processes involved in new protein synthesis, including the availability and assembly of translational machinery. However, this is beyond the scope of this study. Similarly, this model assumes that stable PKC-mRNA is available in the cytosol for new protein synthesis during simulations. Here, we are not attempting to model the transcription of PKC. Our analysis of the PKC life cycle accounts for post-transcriptional events involved in the signaling lifetime of this enzyme. Additionally, we modeled the generation of second-messenger DAG through a brief pulse. This is a simple description of complex processes involved in the generation of second-messenger DAG at the plasma membrane. We are not accounting for all upstream processes such as PIP_2_ and PLC activation prior to the DAG generation at the plasma membrane. The main goal of this study is to analyze the enzymological characteristics of PKC during various phases of its life cycle, not the detailed mechanisms of second-messenger generation and metabolism.

Recent experimental results indicate that PKC adopts an open conformation following second messenger-mediated binding and activation. This state of PKC enzyme is highly sensitive to phosphatases and quickly undergoes PHLPP_1_ and PHLPP_2_ mediated dephosphorylation. The dephosphorylated molecule has reduced thermal stability and, if maintained in this state for long, could be targeted for down-regulation and degradation. Observations also show that hsp70 binds this dephosphorylated species via the turn motif and prevents its association with degradation and down-regulation machinery. Here, our study also analyzes the role of hsp70 during the PKC life cycle especially in rescuing the enzyme from degradation pathways and preventing the down-regulation of active but de-phosphorylated state of the enzyme (Figure 6). Our results show that for endogenous hsp70, a high intensity pulse stimulation of second messenger can lead to complete down-regulation of PKC enzyme (Figures 6A–D: No HSP70 overexpression case). This occurs because high intensity stimulation generates a larger pool of active and dephosphorylated PKC, thus providing enough time for degradation pathways to interact with PKC and target it for down-regulation (Figure 6D: No HSP-60 overexpression case). Our model predicts that if the expression levels of HSP-60/70 are controlled (Figure 6A), it produces a dose-dependent reversal of PKC down-regulation even after the application of a high intensity second messenger stimulation pulse (Figure 6A). We believe this occurs because HSP-60/70 binds with the dephosphorylated PKC molecule and modulates its re-phosphorylation, thus replenishing the enzyme pool in the cytosol. Our results show that heat shock protein overexpression reduces the levels and duration of persistence of the PKC^A^ species (Figure 6D), thus completely or partially restoring the cytosolic enzyme pool.

Interestingly, at first glance the life cycle model of this work suggests the stabilizing roles of PDK1 and HSP70 are functionally equivalent, as both these enzymes are acting on unstable and degradation prone species of PKC isoforms. However, newly synthesized species and dephosphorylated active but mature species are conformationally inequivalent. This model is designed to account for this critical difference, as PDK1 can only bind nascent enzyme. HSP70 is set to bind to mature, active, and dephosphorylated form PKC. This model clearly defines the roles of PDK_1_ and HSP70 during the different phases of the PKC life cycle.

Recent experimental results using a PHLPP knock-down indicate that these novel phosphatase isoforms can act as negative regulators of PKC signaling. Data shows that a PHLPP knockdown in colon cancer cell lines is linked to a 3-fold increase in the expression of PKCβII. Similar trends are also observed in breast epithelial cells. These results suggest that cellular levels of PKC can be modulated by PHLPP isoforms. Our model predicts that blocking PHLPP could reverse the second messenger-induced down-regulation of PKC. Our results also predict that degree of PKC down-regulation could be reversed by inhibiting the PHLPP isoforms in a dose-dependent manner (Figures 7A–D). This occurs because inhibiting the dephosphorylation pathways will reduce the levels of PKC^A^, thus reducing the propensity of down-regulation (Figure 7D). Our results suggest that PHLPP molecules play a critical role in modulating the signaling lifetime of PKC. Targeting the expression or activity of PHLPP’s could be a key factor in fine-tuning the signaling characteristics and functionality of this family of enzymes.

Some of the assumptions we made to construct this model might still be disputed. The enzymological characteristics of cPKCs are critically regulated by their interactions with scaffold proteins. Previous studies indicate that PKCα interacts with scaffold proteins like PICK_1_, PSD95 and SAP97 (Mochly-Rosen et al., 1991; Mochly-Rosen and Gordon, 1998; Gao and Newton 2002; Poole et al., 2004; Larsson, 2006; O'Neill et al., 2011). It seems that interactions with these proteins could be important to the regulation of structure, function, and pharmacological responsiveness of PKC. In the proposed PKC life cycle model, we are not accounting for these interactions. It is possible that, by incorporating these additional interactions, one could further extract more interesting features of the PKC life cycle. These factors could be PKC translocation, anchoring and localization at specific membrane locations. However, it is unlikely that the overall structure and conclusions drawn from this model would be significantly altered. At best, our model is a simplified representation of all the molecular events that occur during the PKC life cycle. Though some kinetic and degradation rates are estimated from previous experimental data, many still are unknown. Therefore, though we are making certain quantitative predictions, further work is needed to precisely estimate all the kinetic rate constants. In parallel, we are also developing a two-compartment model of the PKC life cycle through incorporating membrane translocation and remigration back to cytosol. This version will provide a more realistic representation of the PKC life cycle.

Recent experimental observations suggest that protective action of endogenous heat shock proteins during the PKC lifecycle could be linked to a feed forward regulation (Gao and Newton 2002). These data sets show that phorbol esters, which stimulate the translocation and activation of cPKC family members, may also enhance the responsiveness and expression of heat shock proteins. This raises an interesting possibility that hsp70 could robustly prolong the lifetime of PKC (Gao and Newton 2002). Although activation of PKC targets it for dephosphorylation and down-regulation, the same stimuli may also enhance the capacity of heat shock proteins to rescue PKC from degradation pathways. This could be interesting possibility for modulating the gain-of-function (GOF) and LOF in specific pathological conditions however, in the current study we are not incorporating this feed forward protective cascade in our life cycle model. In our proposed life cycle model, the heat shock protein is considered at a fixed expression and activity levels. In our simulations heat shock protein expression is set at a certain value in the initial conditions and remains constant throughout the simulation. In our upcoming version of this model, we are planning to incorporate a feed forward protective response in a detailed two-compartment system.

The current model is motivated by experimental observations. However, its assumptions as well as its consequences need to be further tested. One of the key assumptions in our proposed model is that only PHLPP_1_ and PHLPP_2_ are responsible for complete dephosphorylation of active PKC. This assumption is supported by observations that PHLPP_1_ and PHLPP_2_ are linked to dephosphorylation of active PKC. However, it is possible that there are other phosphatases present which may also be contributing to enzyme dephosphorylation. Our model only considers PHLPP1 as the relevant phosphatase in the dephosphorylation of active PKC species. Another assumption we made regarding PKC degradation only takes place through dephosphorylated active species. However, some observations show that active and phosphorylated species could also degrade directly at the membrane. Here, we are not incorporating this into our model for the sake of simplicity.

Herein, the model describing the PKC life cycle is based on elementary biochemical reactions. These events are identified and collected from previous experimental observations describing various aspects of PKC lifetime (Keranen et al., 1995; Newton, 1995a; Newton, 1995b; Bornancin and Parker, 1996; Newton, 1996; Edwards and Newton 1997; Keranen and Newton 1997; Newton, 1997; Dutil et al., 1998; Behn-Krappa and Newton 1999; Sonnenburg et al., 2001; Newton, 2003; Violin et al., 2003; Newton, 2004; Chen et al., 2008; Facchinetti et al., 2008; Gould and Newton, 2008; Ikenoue et al., 2008; Steinberg, 2008; Newton, 2010; Antal and Newton, 2013). Models based on elementary biochemical reaction events provide a robust and accurate method to quantify the PKC life cycle as the models derived from basic reaction map are usually free from mathematical artifacts. Once biochemical events describing the PKC life cycle are identified (to reasonable details) then we, quantitatively describe the concentration of various PKC states arising during its lifetime through applying the law of mass action on elementary biochemical reaction steps. The resulting differential equations provide a temporal concentration map of different PKC states and enzyme transformations in response to events like *de novo* synthesis, activation, and termination. These differential equations are then solved through a stiff solver ode15s in MATLAB (Math works 6.4 language of computing). Some of the rate constants in this biochemical reaction system are obtained through limited available experimental data (Bornancin and Parker 1996; Antal and Newton 2013) (Bornancin and Parker 1996; Antal and Newton 2013), for example: 1. Degradation rate of naïve newly synthesized and dephosphorylated species of PKC are set at 0.001 s^−1^, this is based on some observations showing A-638 mutant of PKCα has half-life of less than 10 min and E-638 has half life time of 25 min thus indicating that PKCα enzyme in the absence of carboxylic and activation loop phosphorylations is unstable and can degrade quickly. Similarly, the degradation rate of mature and completely phosphorylated PKC specie is set at 0.00008 s^−1^, this is selected because fully phosphorylated specie of PKC is mature, stable and is protected from phosphatases and proteasome actions and can last for days in cytosol (Keranen et al., 1995; Newton, 1995a; Newton, 1995b; Bornancin and Parker 1996; Newton, 1996; Edwards and Newton 1997; Keranen and Newton 1997; Newton, 1997; Dutil et al., 1998; Behn-Krappa and Newton 1999; Sonnenburg et al., 2001; Newton, 2003; Violin et al., 2003; Newton, 2004; Chen et al., 2008; Facchinetti et al., 2008; Gould and Newton, 2008; Ikenoue et al., 2008; Steinberg, 2008; Newton, 2010; Antal and Newton 2013). The parameter sensitivity map described in supplementary materials (Supplementary Materials 3 Table S2; Supplementary Materials 4 Figure S_13_) show that varying this parameter has almost no effect on overall results of this model. Since the key feature of this model is new synthesis and degradation of PKC enzyme, setting the degradation rates closer to experimentally observed data points can provide some physiological relevance of the PKC model described here; 2. In this model, the dynamics of PDK1-mediated phosphorylation at activation site (P_A_), of PKC is matched closely to experimentally observed data points (Dutil et al., 1998); 3. previous observations suggest that half-life of constitutive PKC autophosphorylations is around 5–10 min (Sonnenburg et al., 2001; Antal and Newton, 2013); 4. PKC is directed towards degradation pathways and PKC degrades quickly in cells lacking mTORC2 (Chen et al., 2008; Facchinetti et al., 2008; Ikenoue et al., 2008). In this model, we set the parameters such that temporal dynamics of PKC degradation, autophosphorylations, and constitutive phosphorylation events are closer to the experimentally observed time scales. In order to study the robustness of model output we also developed parameter sensitivity map (Supplementary Materials 3 Table S2; Supplementary Materials 4 Figures S_1_–S_18_). For this, we selected two cases: first case is of lower intensity second messenger stimulation; second case is of higher intensity second messenger stimulation. For both these cases we studied the effects of 5-fold increase and 90% reduction of each parameter on the total PKC levels. We implemented these sensitivity simulations by selecting and varying each parameter individually and mapping its effects on total PKC levels while all other parameters are fixed at baseline values (Supplementary Materials 2 Table S1). For each parameter four cases i.e., increase and decrease of parameter for lower and higher strength stimulation cases are compared with baseline results and changes in total PKC levels are recorded to establish the relevant effects of that parameter on model output (Supplementary Materials 3 Table S2; Supplementary Materials 4 Figures S_1_–S_18_). Our analysis shows that parameters k_1_, k_15_, k_17_, λ_3_, T are highly sensitive in influencing model output. The parameters k_4_, k_16_, k_18_, k_19_, λ_1_, λ_2_ and PDK1 have only a moderate influence on model results whereas, the parameters k_2_, k_5_, k_6_, k_7_, k_8_, k_9_, k_10_, k_11_, k_12_, k_13_ and k_14_ are not sensitive enough to influence model results. This analysis provides insights that few parameters can drastically affect the model results and though there are still many unknowns regarding the full quantitative description of PKC life cycle the proposed model is robust and may provide a useful tool to study the dynamics of PKC life cycle.

The model results also implies that a constitutively active PDK1 may play a significant and necessary role in the stabilization of newly synthesized, highly labile enzyme. We have also shown that targeting PDK1 expression (Supplementary Materials 4 Figures S_15_) can influence the total PKC levels for both low and high intensity stimulation. We also looked how the changes in PDK1 expression may influence the other enzyme states (Supplementary Materials 4 Figures S_17_). Our results show that reducing PDK1 expression during high intensity stimulation significantly influenced three enzyme states i.e., C.PKC.P_A_.P_H_.P_T_, PKC. P_A_.P_H_.P_T_^A^, and PKC^A^. (Supplementary Materials 4 Figures S_17D,E_).

In the model results presented so far, a 10-min pulse stimulation mimicking the new protein synthesis is only applied at the start of stimulation. This generates sufficient PKC enzyme in the cytosol which can be activated/downregulated by PMA based activation pulse (Figures 2–5). The extent of down-regulation is dependent on the intensity and duration of activation pulse (Figures 2, 3). In connection to this we also asked the question what happens to PKC down-regulation process if another protein synthesis pulse is applied during enzyme down-regulation event? Intriguingly, our results show (Supplementary Materials 4 Figure S_18_) that application of subsequent protein synthesis pulse at 83.33 min during the simulation (Supplementary Materials 4 Figure S_18_) can successfully reverse the down-regulation process and degree of reversal is dependent on the intensity of activation stimulation. For example, for the low intensity activation stimulation (Supplementary Materials 4 Figure S_18_ red solid line (1) and dashed black line (5)) the degree of reversal is largest whereas, for the high intensity stimulation the degree of reversal is moderate (Supplementary Materials 4 Figure S_18_ red solid line (4) and dashed black line (8)). This indicates that in case of high intensity PMA-based activation stimulation significant pool of enzyme has already been lost to degradation pathways.

Intriguing, previous observations show a link between enhanced levels of PKC protein and improved survival in colon cancer (Dowling et al., 2016), pancreatic cancer (Baffi et al., 2019), and non-small-cell lung carcinoma (Halvorsen et al., 2020) proposing an overall tumor suppressive function of PKC. Relating this back to current model the levels of total protein and concentration of protein state C.PKC.P_A_.P_H_.P_T_ could be linked to an overall tumor suppressive function whereas, the process of activation and relative concentrations of PKC.P_A_.P_H_.P_T_^A^, and PKC^A^ can be related to enzyme down-regulation and hence probably these states could have a tumor promoting function.

As mentioned in the introduction in some cases of PKC signaling the fully phosphorylated form of PKC can also be directly targeted for degradation (Parker and Parkinson 2001; Srivastava et al., 2002). To see how that might affect our results we created another version of the basic model (Supplementary Materials 4 Figure S_20_)? In this version we modeled PKC down-regulation (Supplementary Materials 4 Figure S_20_) by two competing pathways: 1) based on dephosphorylated form from cytosol; 2) based on fully phosphorylated form from the membrane. Interestingly, our results (Supplementary Materials 4 Figure S_21_) show that even with two different degradation pathways the temporal dynamics of PKC activation and down-regulation are like the basic model of PKC signaling (Figures 2, 3). This shows that basic model proposed here is robust enough to incorporate some additional biological details of PKC lifetime signaling with little or no variations in the basic temporal profiles.

## Materials and Methods

### Biochemical Reactions

The biochemical reactions that comprise the PKC life cycle (Figure 1) are based on standard mass action kinetics. The law of mass action is the fundamental law of chemical/biochemical transformation. This law describes the rate at which biochemical species such as enzymes, substrates, second messengers interact with each other to form new biochemical products or transform the state of existing biochemical species. This law states that rate of these biochemical transformations is directly proportional to the concentration of participating species. The following set of reactions describes the molecular events that take place during the PKC life cycle. PKC is one of the dynamical variables and represents the naïve form of protein kinase C. PKC-mRNA represents the RNA transcript that codes for the PKC protein. PKC-P_A_ represents the PKC protein that is phosphorylated at an activation site during a PDK_1_ mediated event. C.PKC-P_A_ represents a complex of mTROC_2_ and PKC-P_A;_ whereas the complex C.PKC-P_A_.P_H_.P_T_ represents a PKC complex in which PKC is phosphorylated at all three sites: the activation site, turn site, and the hydrophobic motif site. C.PKC-P_A_.P_H_.P_T_ also represents a form of the enzyme that is inactive, but catalytically competent. This form of the enzyme is stored in the cytosol. The PKC. P_A_.P_H_.P_T_^A^ form of enzyme represents an active, phosphorylated molecule found at the cell membrane. The dephosphorylated, but active species is represented by PKC^A^. The phosphatase, P, is approximated as a fixed parameter rather than a dynamical variable in order, to simplify the simulations. This approximation did not significantly alter the results. PDK_1_ represents the concentration of constitutively active PDK_1_ and is a fixed parameter. Here, ‘T’ represents the concentration of polyribosome and is a fixed parameter. The mTORC_2_, Ca^+2^/DAG/PMA, PHLPP and HSP70 are all fixed parameters and represent the concentrations of complex mTORC2, secondary-messenger DAG, phosphatases PHLPP1 and PHLPP2, and the concentration of heat shock protein 70, respectively.

$$\mathrm{PKC}-\mathrm{mRNA}+T\overset{K_{1}}{\underset{K_{2}}{⇄}}C_{1}$$ (R1)

$$C\overset{K_{3}}{\to }\mathrm{PKC}+T+\mathrm{PKC}-\mathrm{mRNA}$$ (R2)

$$\mathrm{PKC}\overset{\lambda_{1}}{\to }∵$$ (R3)

$$\mathrm{PKC}+\mathrm{PD}K_{1}\overset{K_{4}}{\underset{K_{5}}{⇄}}C_{2}\overset{K_{6}}{\to }C_{\mathrm{PKC}}-P_{A}$$ (R4)

$$\mathrm{PKC-}P_{A}+\mathrm{mTRO}C_{2}\overset{K_{7}}{\underset{K_{8}}{⇄}}\mathrm{PKC}-P_{A}+\mathrm{PD}K_{1}$$ (R5)

$$\mathrm{PKC-}P_{A}\overset{\lambda_{2}}{\to }∵$$ (R6)

$$C_{\mathrm{PKC-}P_{A}}+C_{\mathrm{PKC-}P_{A}}\overset{K_{9}}{\underset{K_{10}}{⇄}}C_{3}\overset{K_{11}}{\to }C_{\mathrm{PKC-}P_{A}P_{H}P_{T}}+C_{\mathrm{PKC-}P_{A}}$$ (R7)

$$C_{\mathrm{PKC-}P_{A}P_{H}P_{T}}+C_{\mathrm{PKC-}P_{A}}\overset{K_{12}}{\underset{K_{13}}{⇄}}C_{4}\overset{K_{14}}{\to }C_{\mathrm{PKC-}P_{A}P_{H}P_{T}}+C_{\mathrm{PKC-}P_{A}P_{H}P_{T}}$$ (R8)

$$C_{\mathrm{PKC-}P_{A}P_{H}P_{T}}+Ca^{\mathrm{2+/DAG}}\overset{K_{15}}{\underset{K_{16}}{⇄}}C^{A}\mathrm{PKC}-P_{A}P_{H}P_{T}$$ (R9)

$$C^{A}\mathrm{PKC}-P_{A}P_{H}P_{T}\overset{K_{17}}{\to }C^{A}\mathrm{PKC}-\mathrm{PHLPP}$$ (R10)

$$C^{A}\mathrm{PKC}\overset{\lambda_{17}}{\to }∵$$ (R11)

$$C_{\mathrm{PKC-}P_{A}P_{H}P_{T}}+\mathrm{HS}P_{\mathrm{60}}+C^{A}PKC\overset{K_{18}}{\underset{K_{19}}{⇄}}C_{5}\overset{K_{20}}{\to }C_{\mathrm{PKC-}P_{A}P_{H}P_{T}}+C_{\mathrm{PKC-}P_{A}P_{H}P_{T}}+\mathrm{HS}P_{60}$$ (R12)

The PKC life cycle model described by the biochemical reaction equations (Figure 1, R_1_-R_12_) can be perturbed either through stimulation of protein synthesis or through secondary-messenger Ca^2+^/DAG-modulated reversible or PMA-modulate irreversible activation. New PKC synthesis is described by equations R_1_ and R_2_. (Figure 1). These reactions show that when new protein synthesis is stimulated, the PKC transcript is loaded with polyribosome “T,” as described by R_1_ (Figure 1). The loaded transcript “C_1_” can then undergo translation to generate additional PKC protein (Figure 1). During induction, the signals “k_3_” from multiple upstream kinases act on this loaded transcript and initiate the *de-novo* synthesis of naïve PKC from its transcript (Figure 1). This translation event is described by the biochemical reaction event R_2_ (Figure 1). The naïve PKC molecule is highly unstable and can quickly degrade via degradation pathways, as described by the biochemical reaction event R_3_ (Figure 1). However, the naïve, unstable PKC molecule has a strong affinity for constitutively active kinases (i.e., PDK_1_), and undergoes maximum phosphorylation at its activation site (i.e., P_A_) as a result (Figure 1, reaction event R_4_). The phosphorylated enzyme (PKC.P_A_) forms a biochemical complex (C.PKC.P_A_) with the mTORC_2_ complex (Figure 1, reaction event R_5_). Phosphorylation at activation site P_A_ reduces the degradation rate of naïve enzyme because PKC.P_A_ is more stable and degrades more slowly as described by R_6_. In contrast, λ_2_ represents the degradation rate of this enzyme species (Figure 1). The C.PKC.P_A_ complex stimulates autophosphorylation at the hydrophobic sites of the PKC enzyme. This process generates the fully mature, auto-inhibited, catalytically competent species C.PKC.P_A_.P_H_.P_T_. These autophosphorylation events are described through equations R_7_ & R_8_ (Figure 1). The inactive, auto-inhibited, catalytically competent species is stored at different locations in the cytosol. Following membrane receptor stimulation, second messengers prompt this species to migrate from the cytosol to the membrane, where a second messenger activates it (Figure 1, reaction event R_9_). The active C.PKC.P_A_.P_H_.P_T_^A^ form of the enzyme is prone to rapid dephosphorylation by phosphatases PHLPP_1_ and PHLPP_2_. These dephosphorylation events are described by equation R_10_ (Figure 1). Upon dephosphorylation, the active form of the enzyme, C.PKC^A^, is prone to degradation, and is thus targeted by degradation machinery for removal (Figure 1, reaction event R_11_). Heat shock proteins HSP-60 and HSP-70 act on the active and dephosphorylated form of the enzyme, rescuing and recovering PKC from degradation pathways and restoring the pool of inactive, catalytically competent PKC in the cytosol (Figure 1, reaction event R_12_).

### Induction

During simulations, the induction of new proteins was simulated by a 10-min pulse of protein synthesis, which produced an increase in the total PKC protein concentration.

### Temporal Dynamics

The law of mass action as described in discussion section and above in *Biochemical Reactions*, is used to generate differential equations through accounting the mass balance of each biochemical specie as described in above equations R_1_-R_12_. The differential equations provide the temporal variations in the concentration of each specie participating in above reaction network i.e., R_1_-R_12_. The differential equations (Supplementary Material 5) resulting from above interactions were integrated through nonlinear solvers using MATLAB (MathWorks-MATLAB; Scientific Computing). Matlab, is a matrix-based language of scientific computing which allows the most natural expression of computational mathematics. Here, “ode15s” is used to solve the differential equations in Matlab. The solver “ode15s” is based on implicit method and demonstrates robust performance for most stiff system of equations. A system of differential equations describing reaction network R_1_-R_12_ could be stiff as some solutions may vary slowly but some other solutions might vary rapidly. Some of the dynamical coefficients’ values were estimated from experimental data such as degradation rates, and rates of constitutive and autophosphorylations. Unknown rate constants were scaled to obtain dynamics that were comparable to experimental values (Keranen et al., 1995; Newton, 1995a; Newton, 1995b; Bornancin and Parker, 1996; Newton, 1996; Edwards and Newton 1997; Keranen and Newton 1997; Newton, 1997; Dutil et al., 1998; Behn-Krappa and Newton 1999; Sonnenburg et al., 2001; Newton, 2003; Violin et al., 2003; Newton, 2004; Chen et al., 2008; Facchinetti et al., 2008; Gould and Newton, 2008; Ikenoue et al., 2008; Steinberg, 2008; Newton, 2010; Antal and Newton, 2013). Unless otherwise stated, all of the molecular concentrations in the model are expressed as ng/ml and time is represented in minutes.

## Data Availability

The original contributions presented in the study are included in the article/Supplementary Material, further inquiries can be directed to the corresponding author. The code files used in the simulation are freely available at https://datadryad.org/stash/share/8xh1K2GnbNGQdLy_Xf8zJdDLHiXgoH6Ef-u7ns5Tq8I. The DOI is: https://doi.org/10.5061/dryad.sqv9s4n08.
