# Supplementary material for: Protein Kinase C Life Cycle: Explained Through Systems Biology Approach
Source: Front Physiol. 2022 Apr 14;13:818688. doi: 10.3389/fphys.2022.818688 (PMC9049586; doi:10.3389/fphys.2022.818688)

Supplementary Material 1: Supplementary Figures:

Supplementry Figure Captions:

Figure S_1: The numerical simulations comparing the down-regulation characteristics of cPKC during sequential application of three lower intensity and higher intensity second messenger pulse stimulations. These results indicate that initial application of a 10-minute protein synthesis pulse leads to the generation of a naïve, and unstable enzyme which is quickly stabilized through constitutive phosphorylation events at the activation, turn and hydrophobic sites. Once stable and phosphatase/proteasome resistant this inactive form of cPKC is stored in cytosol. Here, dashed red lines indicate stimulation (protein synthesis as well as second-messenger) and blue solid lines indicate non-stimulated condition. (a) Total PKC enzyme during protein synthesis and second messenger mediated stimulation. These results show that application of a brief pulse (**dashed-line region**) shows the quick generation of cPKC enzyme and stabilization. These results also show that at later time points (i.e., 50, 150 and 200 minutes in the stimulation) the application of three lower intensity and three higher intensity second messenger pulse (15-minute pulse mimicking Ca^{+2}/DAG: lower intensity DAG is set at 0.0005 nM and higher intensity DAG is set at 0.05nM) leads to the down-regulation of cPKC. However, the degree of down-regulation in case of lower intensity stimulation is minimal in contrast, degree of down-regulation is quite significant in case of higher intensity stimulation. (b) concentration of naïve, newly synthesized unstable form of cPKC. (c) concentration of PKC species phosphorylated at activation site. (d) concentration of PKC complex $C.PKCP_A$. (e) concentration of PKC complex, phosphorylated at all three sites i.e., activation, turn and hydrophobic sites. (f) concentration of active complex $PKC.P_A.P_H.P_T.^A$ after second messenger binding. Again, these results indicate that activation levels of this complex are dependent on the levels of second-messenger stimulation. (g) concentration of dephosphorylated but active PKC^A molecule.

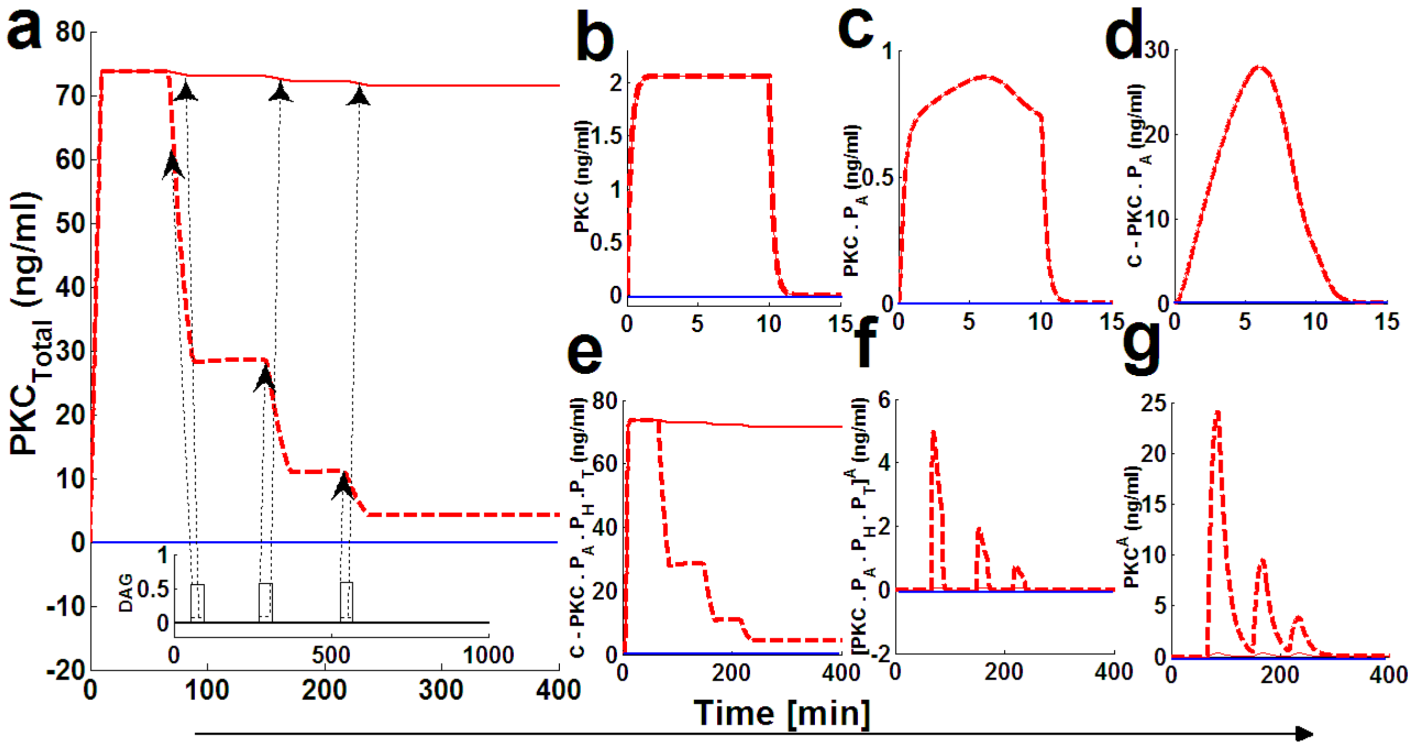

a
b
c
d
e
f
g
PKC$_{Total}$ (ng/ml)
PKC (ng/ml)
PKC . P$_A$ (ng/ml)
C - PKC . P$_A$ (ng/ml)
C - PKC . P$_A$. P$_H$. P$_T$ (ng/ml)
[PKC . P$_A$. P$_H$. P$_T$]^A (ng/ml)
PKC^A (ng/ml)
DAG
Time [min]

Figure S_1:

Supplementary Material 2: Table 1 Numerical values of biochemical rate parameters representing the molecular model describing the PKC life cycle:

**Parameter**	**Description**	**Numerical Values**
k_1	kinetic rate constant describing the loading of PKC transcript with translation machinery	0.05 nM^{-1} sec^{-1}
k_2	kinetic rate constant describing the unloading of PKC transcript with translation machinery	0.001 sec^{-1}
k_3	Translation rate constant of PKC	3.0 sec^{-1}
k_4	Association rate constant of PDK1-PKC binding.	0.12 nM^{-1} sec^{-1}
k_5	Dissociation rate constant of complex C_2	0.005 sec^{-1}
k_6	Rate constant for the phosphorylation of PKC at activation loop	0.45 sec^{-1}
k_7	Association rate constant of PKC with mTROC2 complex	0.35 nM^{-1} sec^{-1}
k_8	Dissociation rate constant of complex C.PKC.P_A	0.0012 sec^{-1}
k_9	Association rate constant of C.PKC.P_A complex with its neighbor	0.0032 nM^{-1} sec^{-1}
k_{10}	Dissociation rate constant of complex C_3	1 sec^{-1}
k_{11}	Phosphorylation rate constant at hydrophobic and turn motif of PKC enzyme due to neighbor-neighbor interactions.	0.005 sec^{-1}
k_{12}	Association rate constant of C.PKC.P_A complex with C.PKC.P_A.P_H.P_T due to auto-phosphorylation events	0.01 nM^{-1} sec^{-1}
k_{13}	Dissociation rate constant of complex C4	1 sec^{-1}
k_{14}	Phosphorylation rate constant at hydrophobic and turn motif of	0.06 sec^{-1}

	PKC enzyme due to autophosphorylation interactions.	
k_{15}	Second messenger binding and activation of $C.PKC.P_A.P_H.P_T{}^A$ at the membrane	0.02 nM^{-1} sec^{-1}
k_{16}	Dissociation rate constant of $C.PKC.P_A.P_H.P_T$ at the membrane	0.002 sec^{-1}
k_{17}	Rate constant of PHLPP mediated dephosphorylation of $C.PKC.P_A.P_H.P_T{}^A$ at the membrane	0.01 sec^{-1}
k_{18}	Association rate of hsp60/70, $C.PKC^A$ and $C.PKC.P_A.P_H.P_T$	0.01 nM^{-1} sec^{-1}
k_{19}	Dissociation rate constant of complex C_5	0.12*30 sec^{-1}
k_{20}	Rate constant for re-phosphorylation of PKC	0.01 sec^{-1}
PDK1	Concentration of PDK1	0.5 nM
T	Concentration of Translational machinery for new synthesis of enzyme	0.05 nM
mTORC2	Concentration of mTORC2 complex	0.5 nM
PKC mRNA	Concentration of PKC transcript	50 nM
λ_1	Degradation rate of newly synthesized, naïve, unphosphorylated cPKC molecule	0.001 sec^{-1}
λ_2	Degradation rate of cPKC molecule phosphorylated at activation site	0.00008 sec^{-1}
λ_3	Degradation rate of dephosphorylated but active PKC^A molecule	0.001 sec^{-1}

Supplementary Material 3: Table 2: Parameter sensitivity map of biochemical rate parameters representing the molecular model describing the PKC life cycle:

No	Parameter	Low Second-messenger stimulation case 5-fold increase	Low Second-messenger stimulation case 90% decrease	High Second-messenger stimulation case 5-fold increase	High Second-messenger stimulation case 90% decrease
1.	k_1	400% increase in total PKC levels	80% decrease in total PKC levels	400% increase in total PKC levels (only transiently first 50 minutes)	80% decrease in total PKC levels (only transiently first 50 minutes)
2.	k_2	No change from baseline results	No change from baseline results	No change from baseline results	No change from baseline results
3.	k_4	2% increase in total PKC levels	10% decrease in total PKC levels	2% increase in total PKC levels (only transiently first 50 minutes)	10% decrease in total PKC levels (only transiently first 50 minutes)
4.	k_5	No change from baseline results	No change from baseline results	No change from baseline results	No change from baseline results
5.	k_6	No change from baseline results	No change from baseline results	No change from baseline results	No change from baseline results
6.	k_7	No change from baseline results	No change from baseline results	No change from baseline results	No change from baseline results
7.	k_8	No change from baseline results	No change from baseline results	No change from baseline results	No change from baseline results
8.	k_9	No change from baseline results	No change from baseline results	No change from baseline results	No change from baseline results
9.	k_{10}	No change from baseline results	No change from baseline results	No change from baseline results	No change from baseline results
10.	k_{11}	No change from baseline results	No change from baseline results	No change from baseline results	No change from baseline results
11.	k_{12}	No change from baseline results	No change from baseline results	No change from baseline results	No change from baseline results
12.	k_{13}	No change from baseline results	No change from baseline results	No change from baseline results	No change from baseline results

13.	k_{14}	No change from baseline results	No change from baseline results	No change from baseline results	No change from baseline results
14.	k_{15}	5% decrease in total PKC levels	1% increase in total PKC levels	0.5% decrease in total PKC levels (steady state levels)	35-fold increase in total PKC levels (steady state levels)
15.	k_{16}	No change from baseline results	No change from baseline results	4% increase in total PKC levels (steady state)	No change from baseline results
16	k_{17}	No change from baseline results	No change from baseline results	No change from baseline results	8-fold increase in total PKC levels (steady state levels)
17	k_{18}	No change from baseline results	No change from baseline results	2.5-fold increase in total PKC levels (steady state levels)	30% decrease in total PKC levels (steady state levels)
18	k_{19}	No change from baseline results	No change from baseline results	20% decrease in total PKC levels	5-fold increase in total PKC levels
19	k_{20}	No change from baseline results	No change from baseline results	3-fold increase in total PKC levels	10% decrease in total PKC levels
20	λ1	10% decrease in total PKC levels	2% increase in total PKC levels	10% decrease in total PKC levels (only transiently first 50 minutes)	2% increase in total PKC levels (only transiently first 50 minutes)
21	λ2	No change from baseline results	No change from baseline results	No change from baseline results	No change from baseline results
22	λ3	No change from baseline results	No change from baseline results	3% decrease from baseline	15-fold increase from baseline results
23	PDK1	2% increase in total PKC levels	15% decrease in total PKC levels	2% increase in total PKC levels (only transiently first 50 minutes)	15% decrease in total PKC levels (only transiently first 50 minutes)
24	T	400% increase in total PKC levels	80% decrease in total PKC levels	400% increase in total PKC levels (only transiently first 50 minutes)	80% decrease in total PKC levels (only transiently first 50 minutes)

Supplementary Material 4: Parameter Sensitivity Figures as related to table 2:

Figure S_2: The effect of parameter k_1 on the levels of PKC_{Total} simulations with 5-times increase in k_1 and 90% reduction in k_1 are compared with baseline simulations (parameters set at values of table1). (a) case of lower intensity simulation. (b) case of higher intensity simulation.

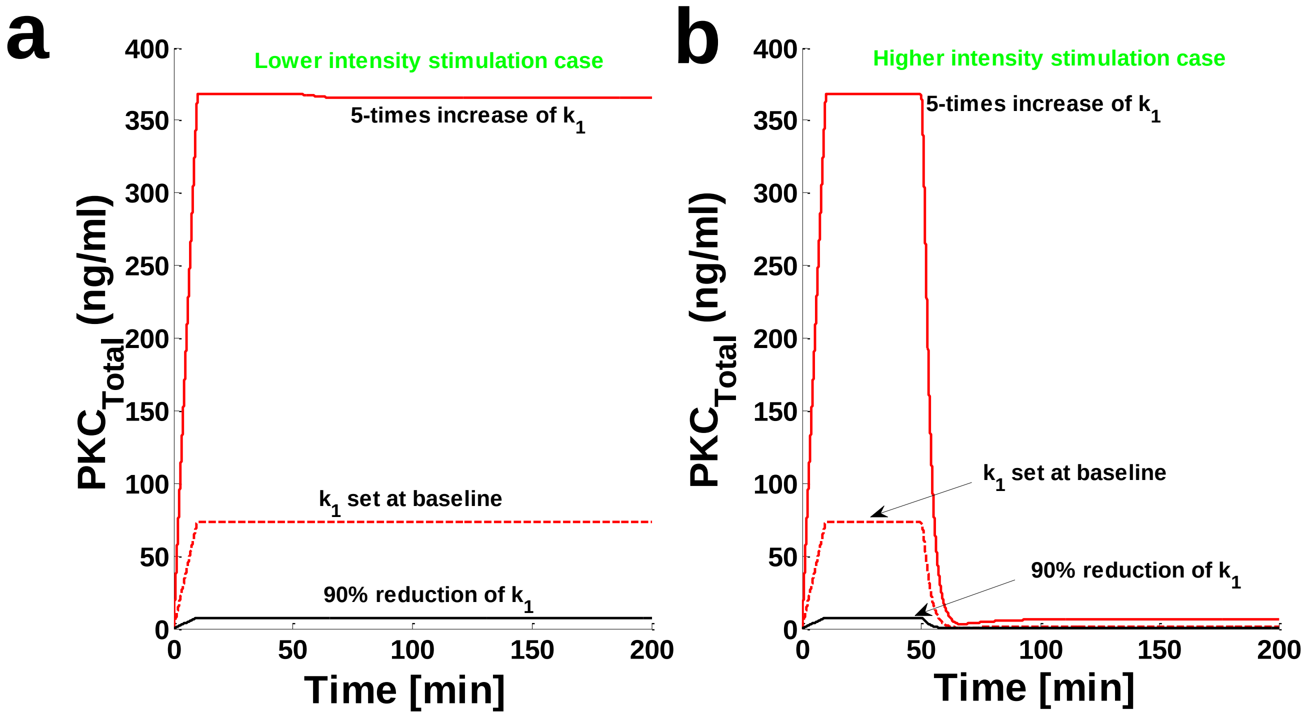

Figure S3: The effect of parameter k_1 on the levels of PKC_{Total} simulations with 5-times increase in k_2 and 90% reduction in k_2 are compared with baseline simulations (parameters set at values of table1). (a) case of lower intensity simulation. (b) case of higher intensity simulation.

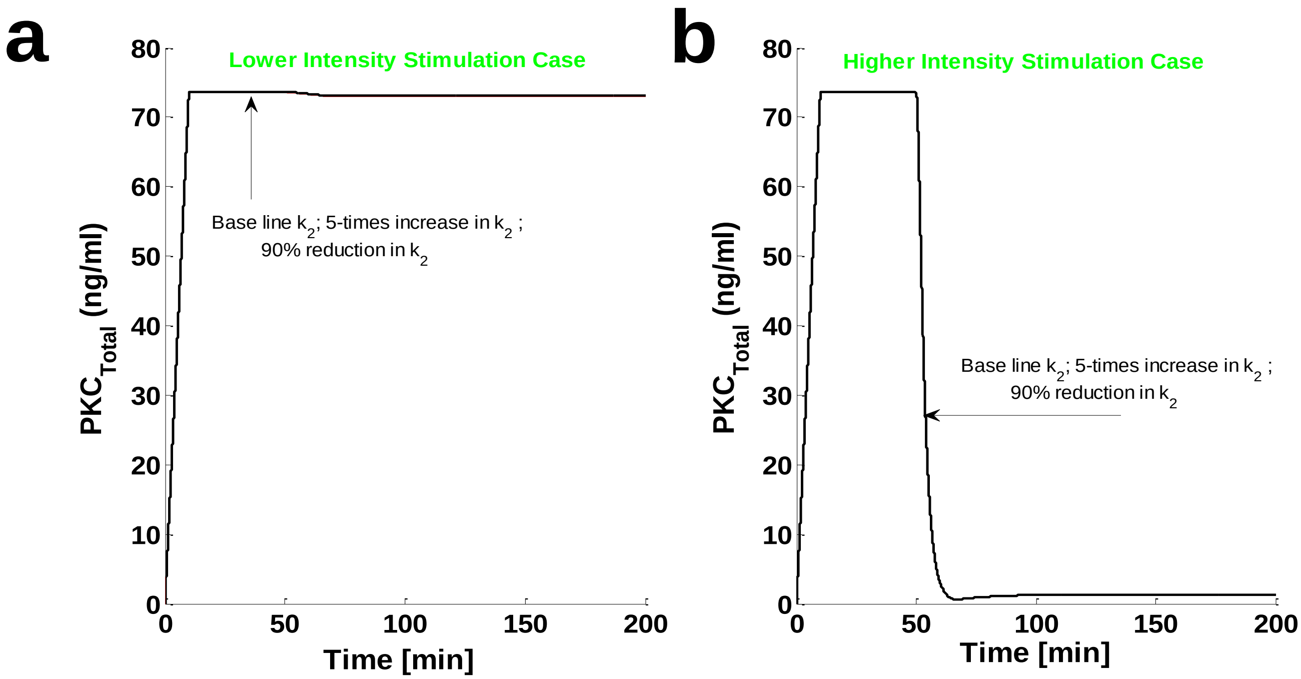

Figure S_4: The effect of parameter k_4 on the levels of PKC_{Total} simulations with 5-times increase in k_4 and 90% reduction in k_4 are compared with baseline simulations (parameters set at values of table1). (a) case of lower intensity simulation. (b) case of higher intensity simulation.

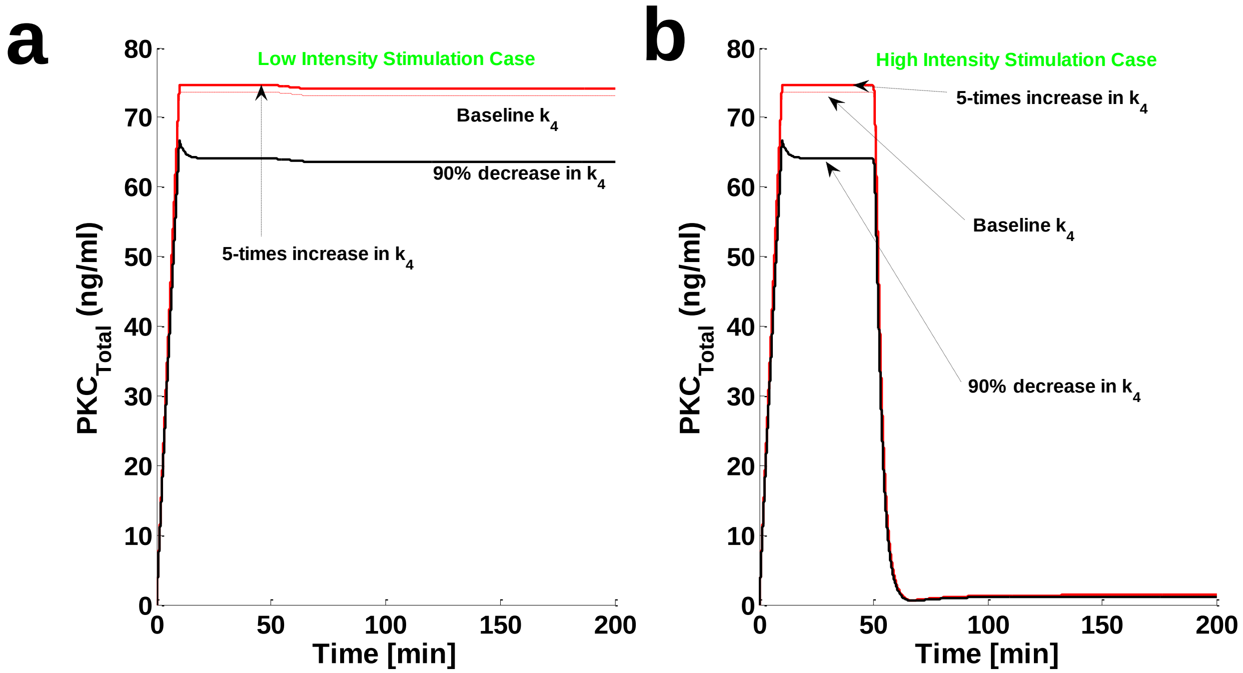

Figure S_5: The effect of parameter k_5 on the levels of PKC_{Total} simulations with 5-times increase in k_5 and 90% reduction in k_5 are compared with baseline simulations (parameters set at values of table1). (a) case of lower intensity simulation. (b) case of higher intensity simulation.

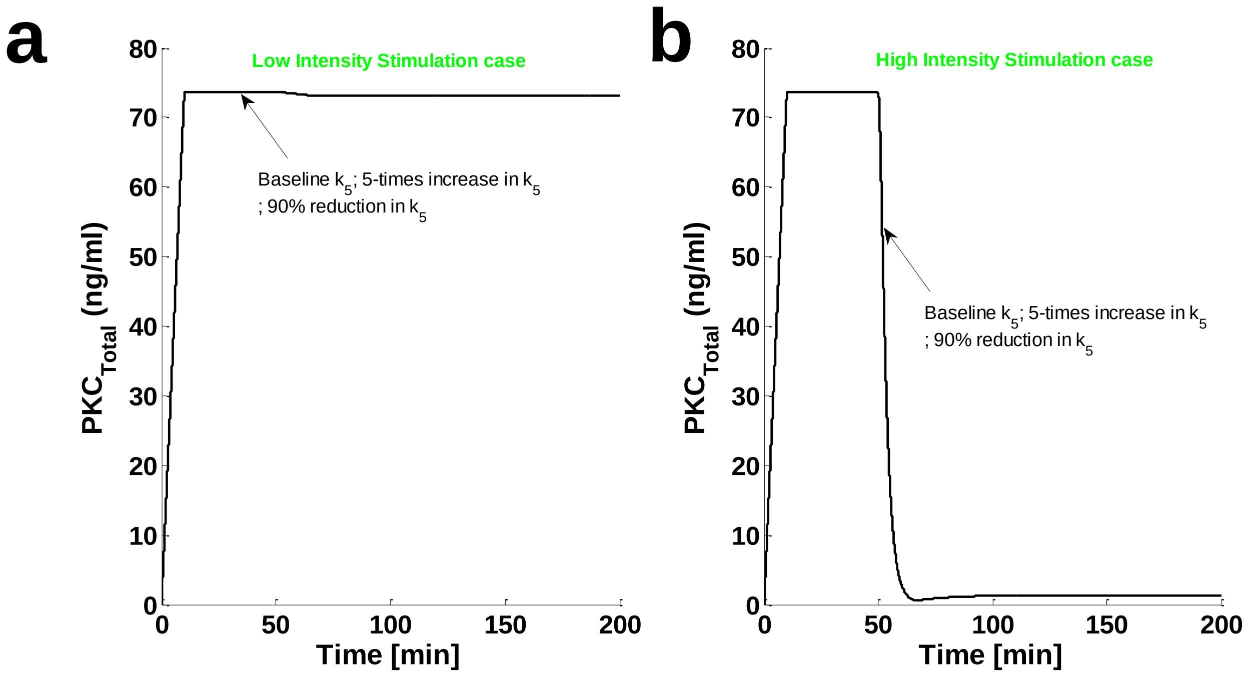

Figure S_6: The effect of parameter k_6 on the levels of PKC_{Total} simulations with 5-times increase in k_6 and 90% reduction in k_6 are compared with baseline simulations (parameters set at values of table1). (a) case of lower intensity simulation. (b) case of higher intensity simulation.

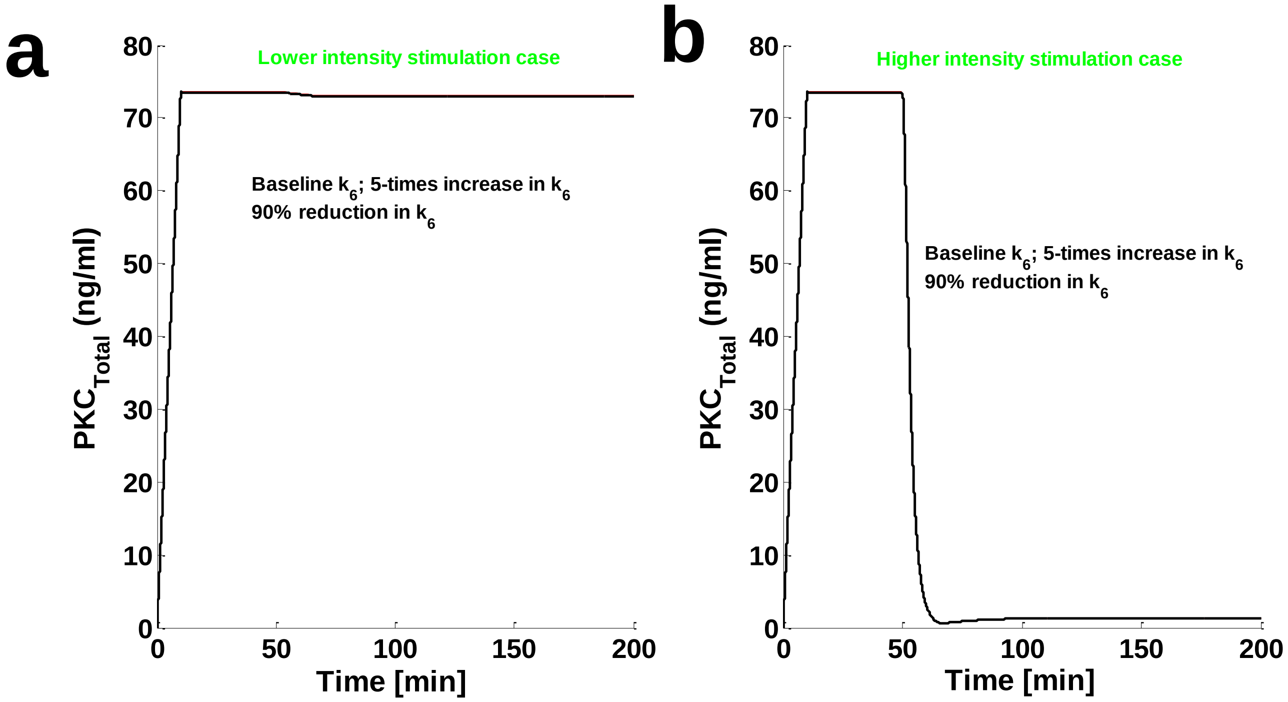

Figure S_7: The effect of parameter k_{15} on the levels of PKC_{Total} simulations with 5-times increase in k_{15} and 90% reduction in k_{15} are compared with baseline simulations (parameters set at values of table1). (a) case of lower intensity simulation. (b) case of higher intensity simulation.

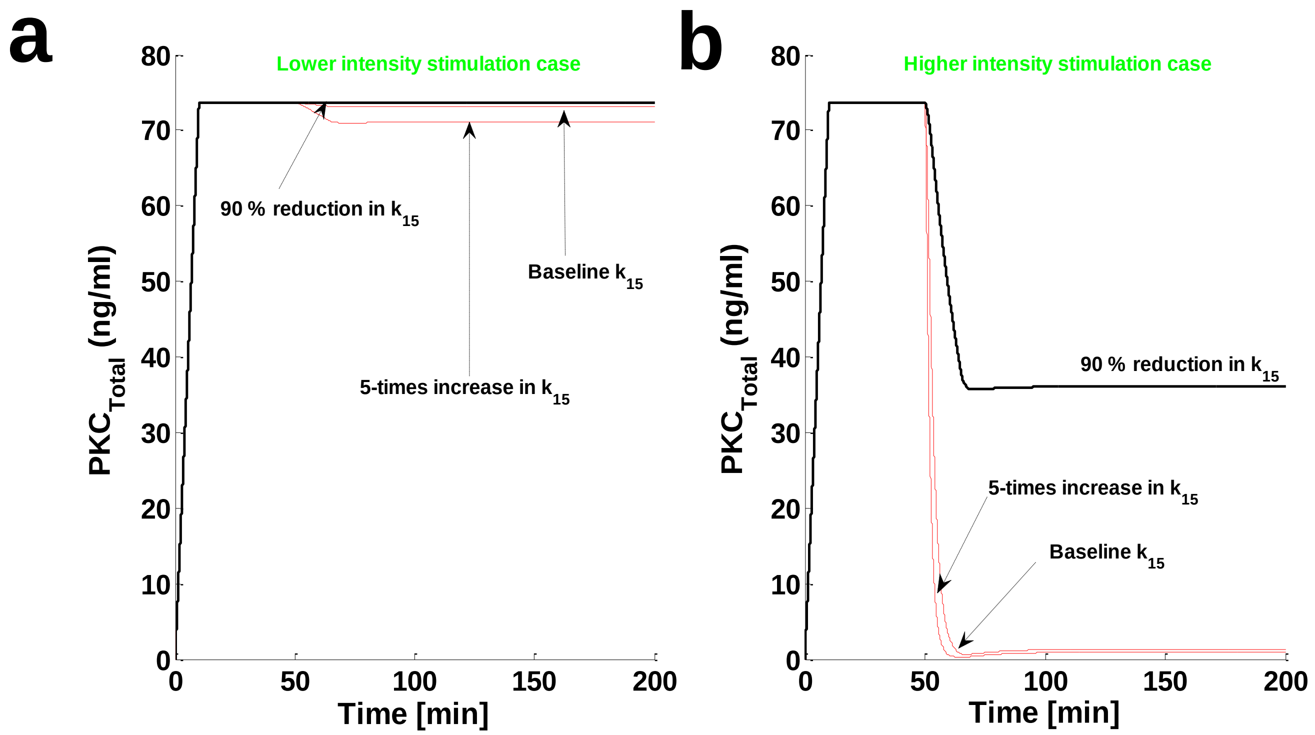

Figure S_8: The effect of parameter k_{16} on the levels of PKC_{Total} simulations with 5-times increase in k_{16} and 90% reduction in k_{16} are compared with baseline simulations (parameters set at values of table1). (a) case of lower intensity simulation. (b) case of higher intensity simulation.

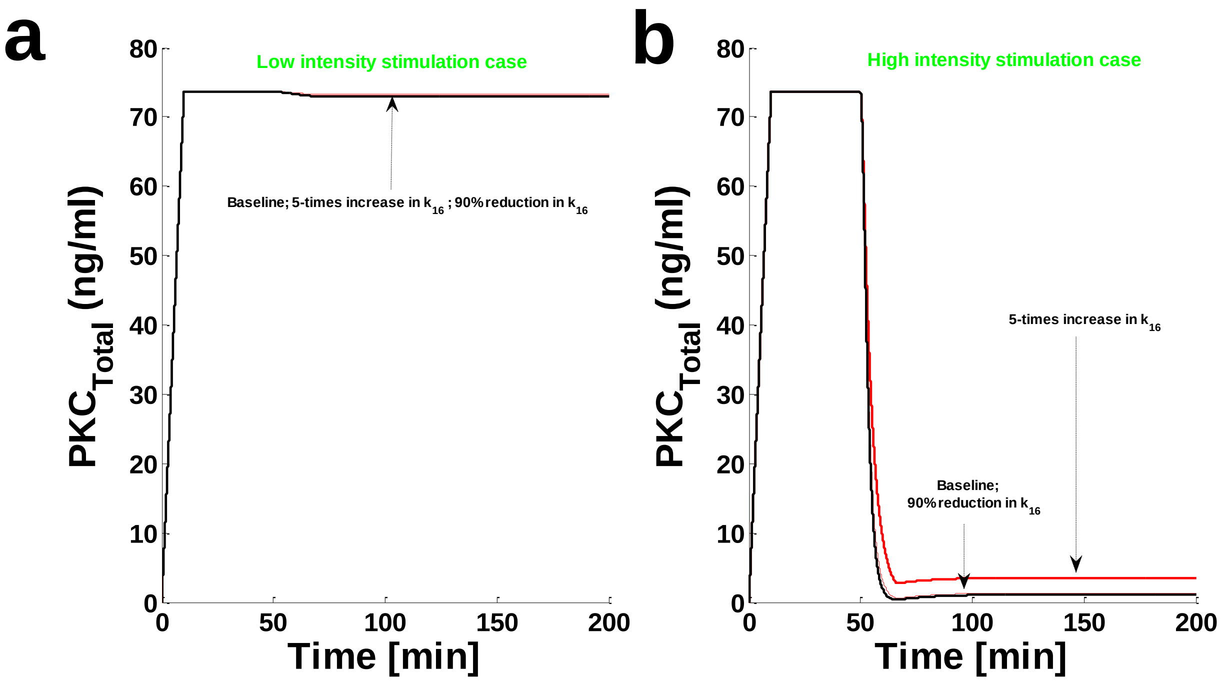

Figure S_9: The effect of parameter k_{17} on the levels of PKC_{Total} simulations with 5-times increase in k_{17} and 90% reduction in k_{17} are compared with baseline simulations (parameters set at values of table1). (a) case of lower intensity simulation. (b) case of higher intensity simulation.

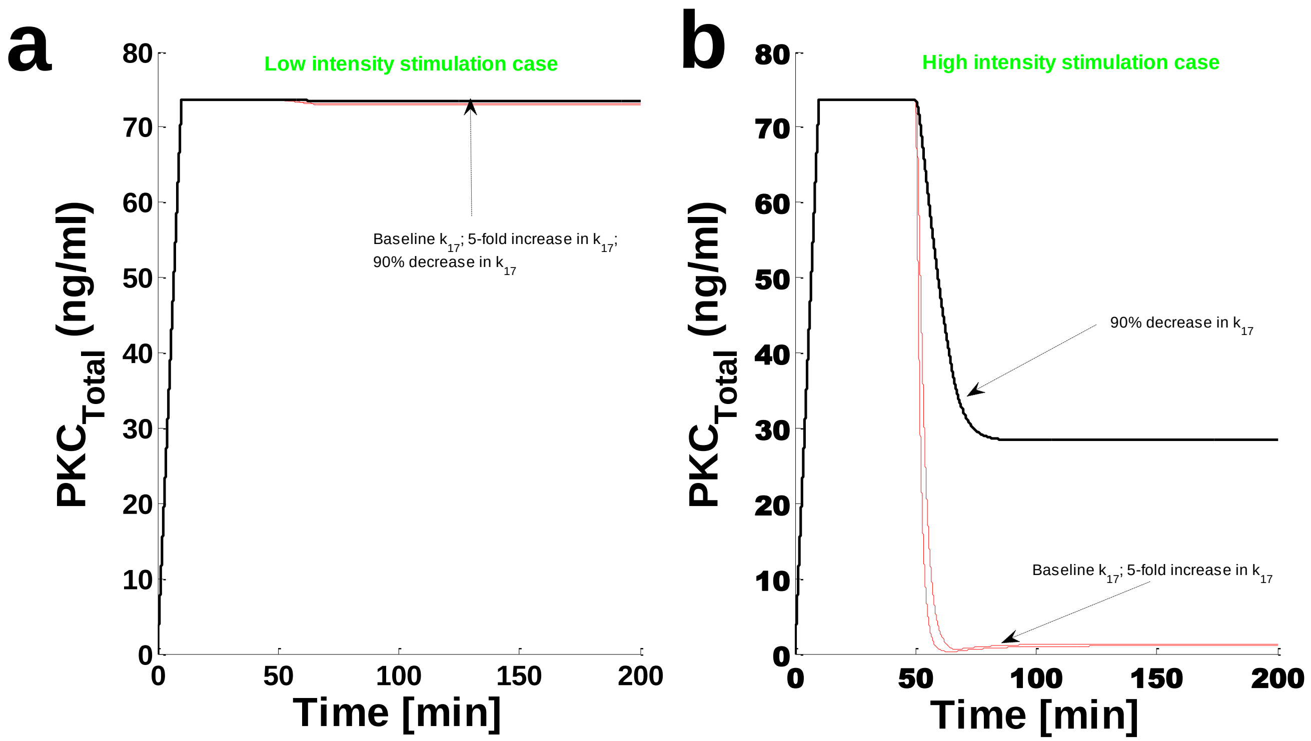

Figure S_{10}: The effect of parameter k_{18} on the levels of PKC_{Total} simulations with 5-times increase in k_{18} and 90% reduction in k_{18} are compared with baseline simulations (parameters set at values of table1). (a) case of lower intensity simulation. (b) case of higher intensity simulation.

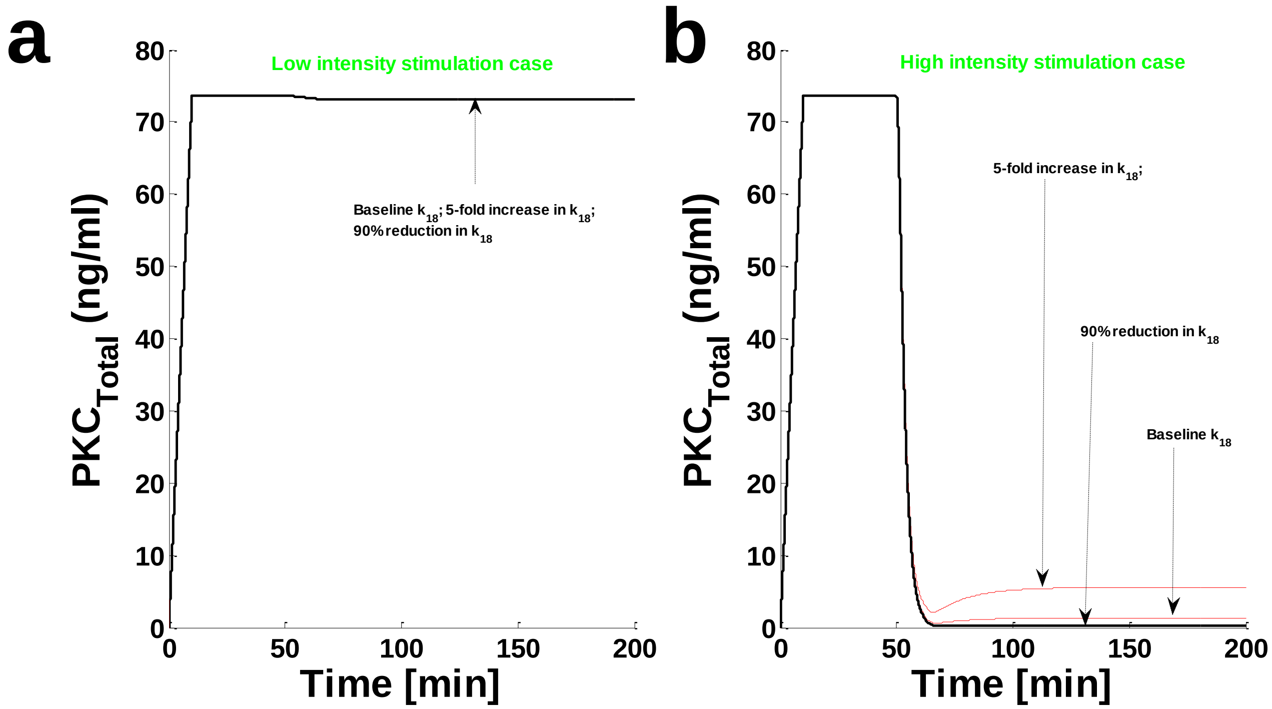

Figure S_{11}: The effect of parameter k_{19} on the levels of PKC_{Total} simulations with 5-times increase in k_{19} and 90% reduction in k_{19} are compared with baseline simulations (parameters set at values of table1). (a) case of lower intensity simulation. (b) case of higher intensity simulation.

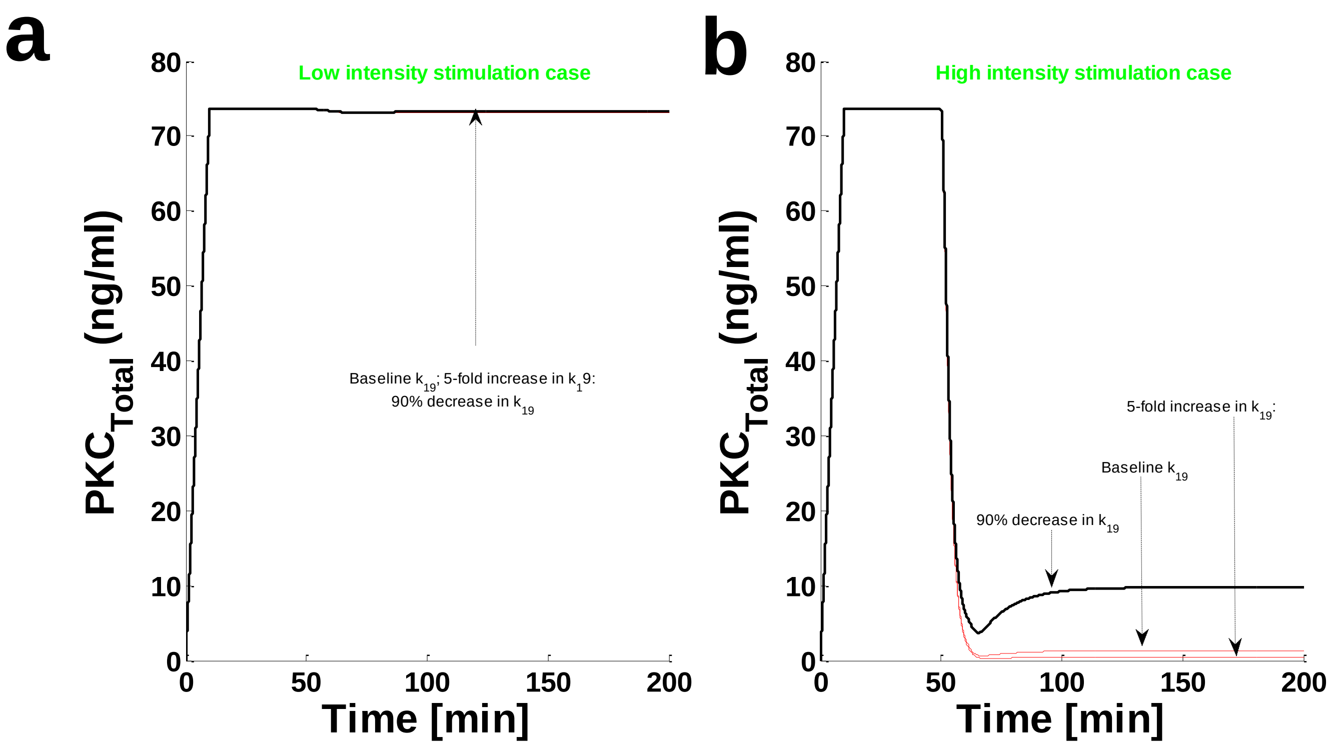

Figure S_{12}: The effect of parameter λ_1 on the levels of PKC_{Total} simulations with 5-times increase in λ_1 and 90% reduction in λ_1 are compared with baseline simulations (parameters set at values of table1). (a) case of lower intensity simulation. (b) case of higher intensity simulation.

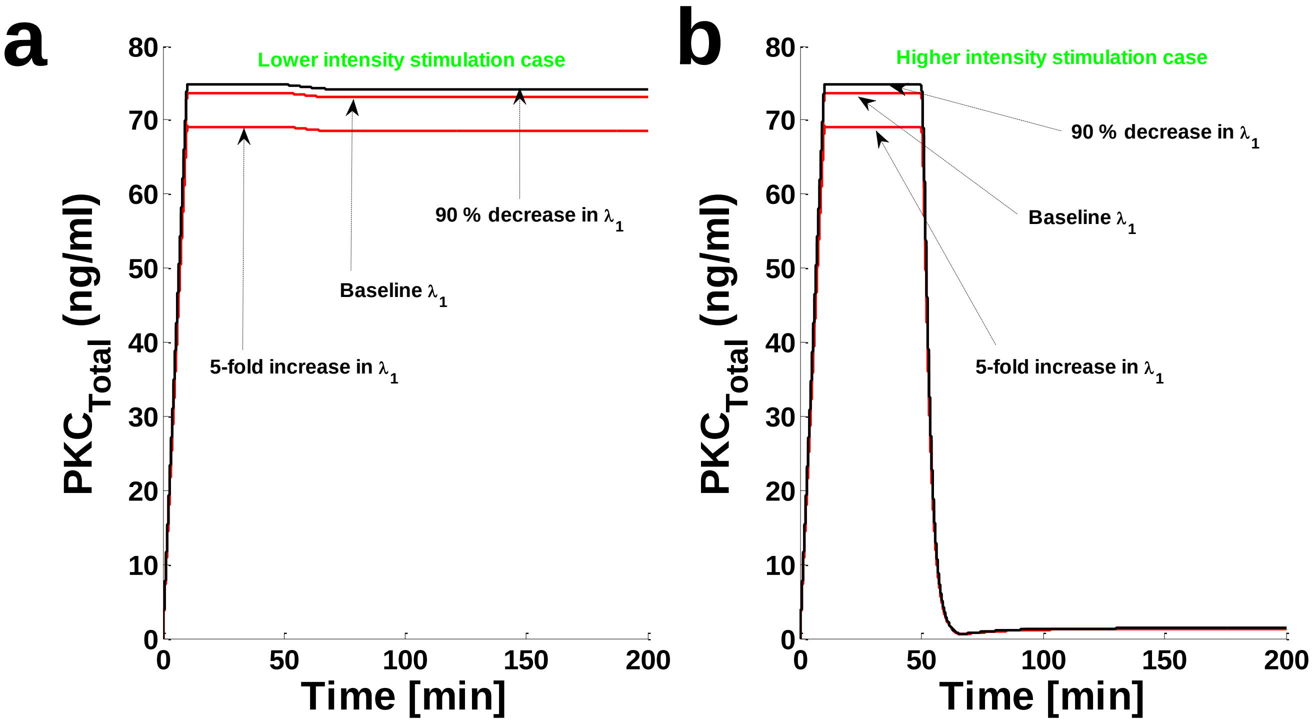

Figure S13: The effect of parameter λ_2 on the levels of PKC_{Total} simulations with 5-times increase in λ_2 and 90% reduction in λ_2 are compared with baseline simulations (parameters set at values of table1). (a) case of lower intensity simulation. (b) case of higher intensity simulation.

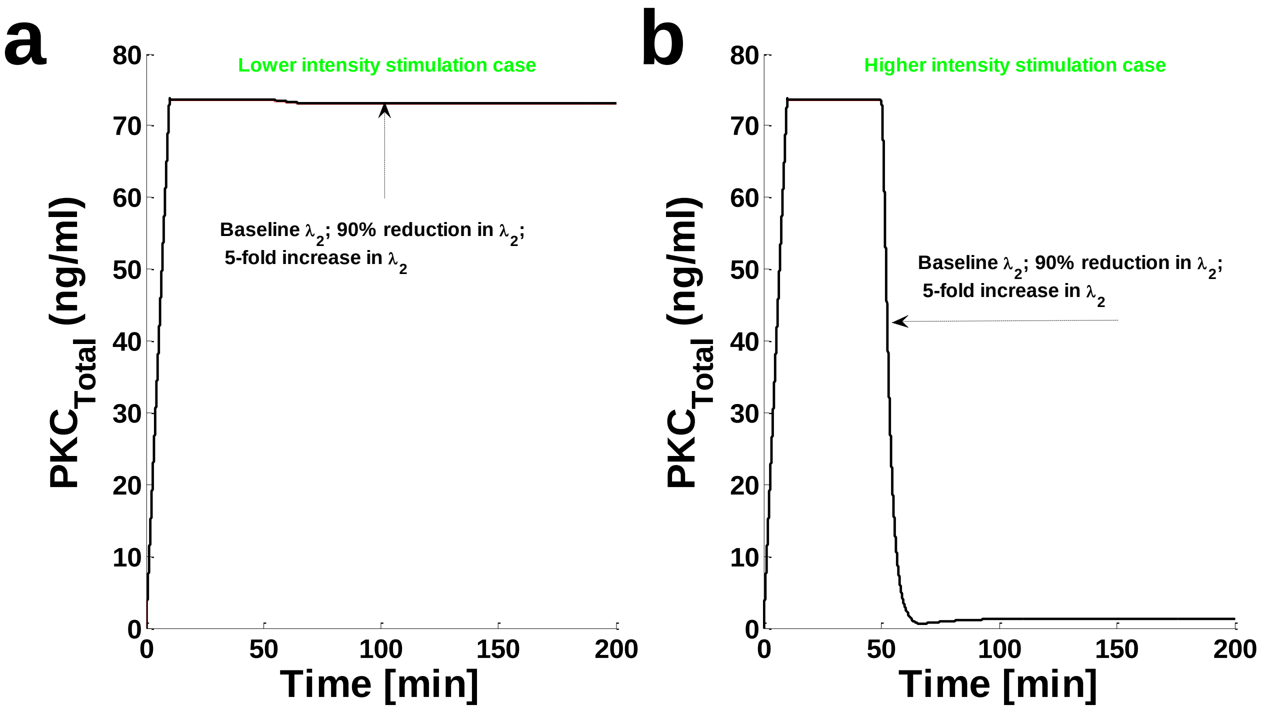

Figure S_{14}: The effect of parameter λ_3 on the levels of PKC_{Total} simulations with 5-times increase in λ_3 and 90% reduction in λ_3 are compared with baseline simulations (parameters set at values of table1). (a) case of lower intensity simulation. (b) case of higher intensity simulation.

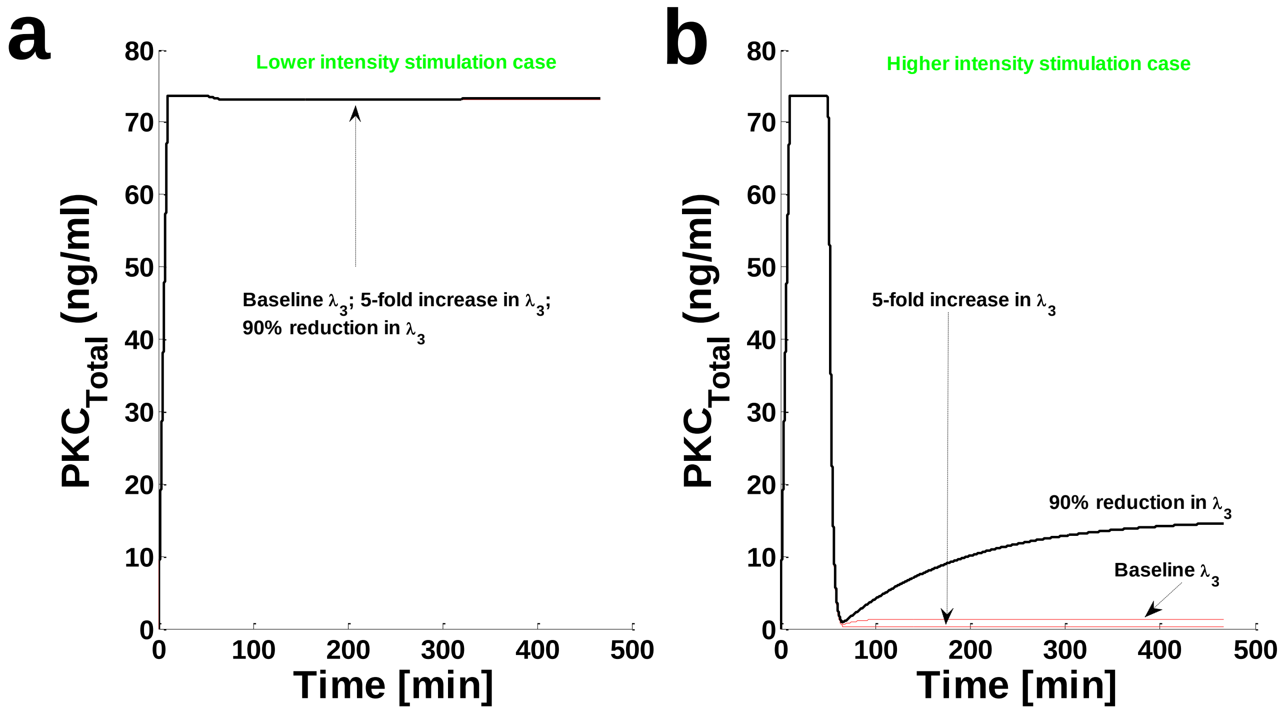

Figure S_{15}: The effect of parameter PDK_1 on the levels of PKC_{Total} simulations with 5-times increase in PDK_1 and 90% reduction in PDK_1 are compared with baseline simulations (parameters set at values of table1). (a) case of lower intensity simulation. (b) case of higher intensity simulation.

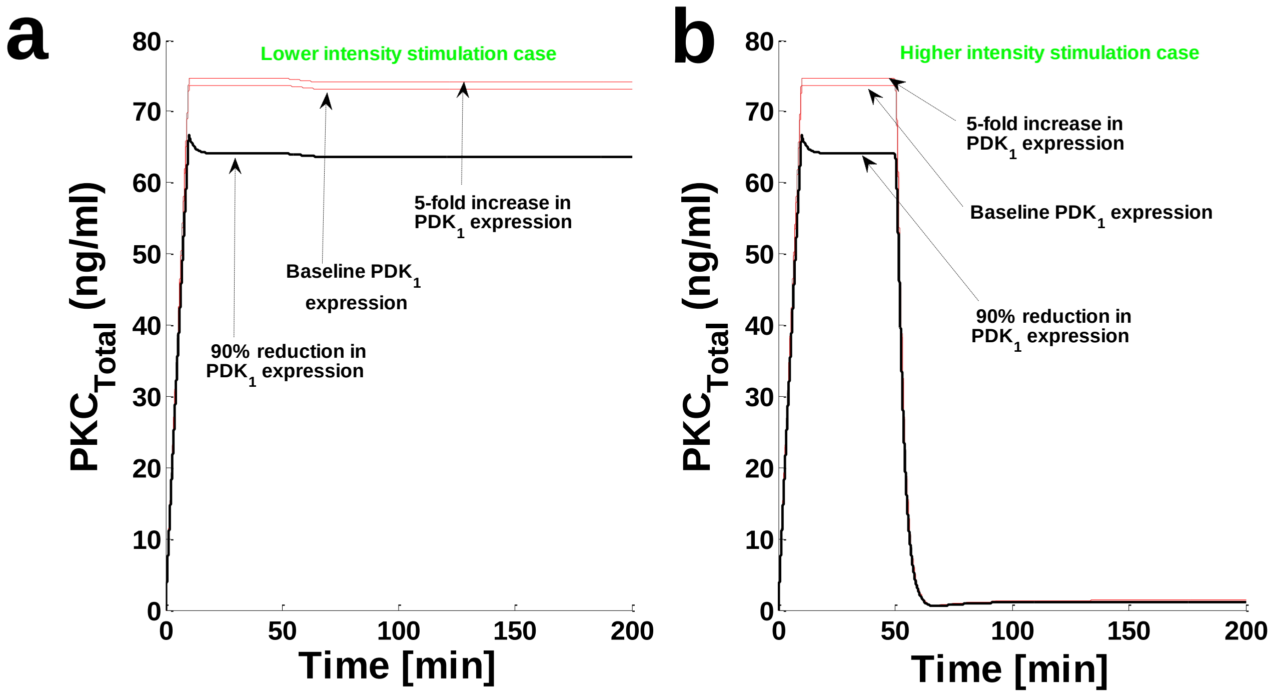

Figure S_{16}: The effect of parameter mTORC2 on the levels of PKC_{Total} simulations with 5-times increase in mTORC2 and 90% reduction in mTORC2 are compared with baseline simulations (parameters set at values of table1). (a) case of lower intensity simulation. (b) case of higher intensity simulation.

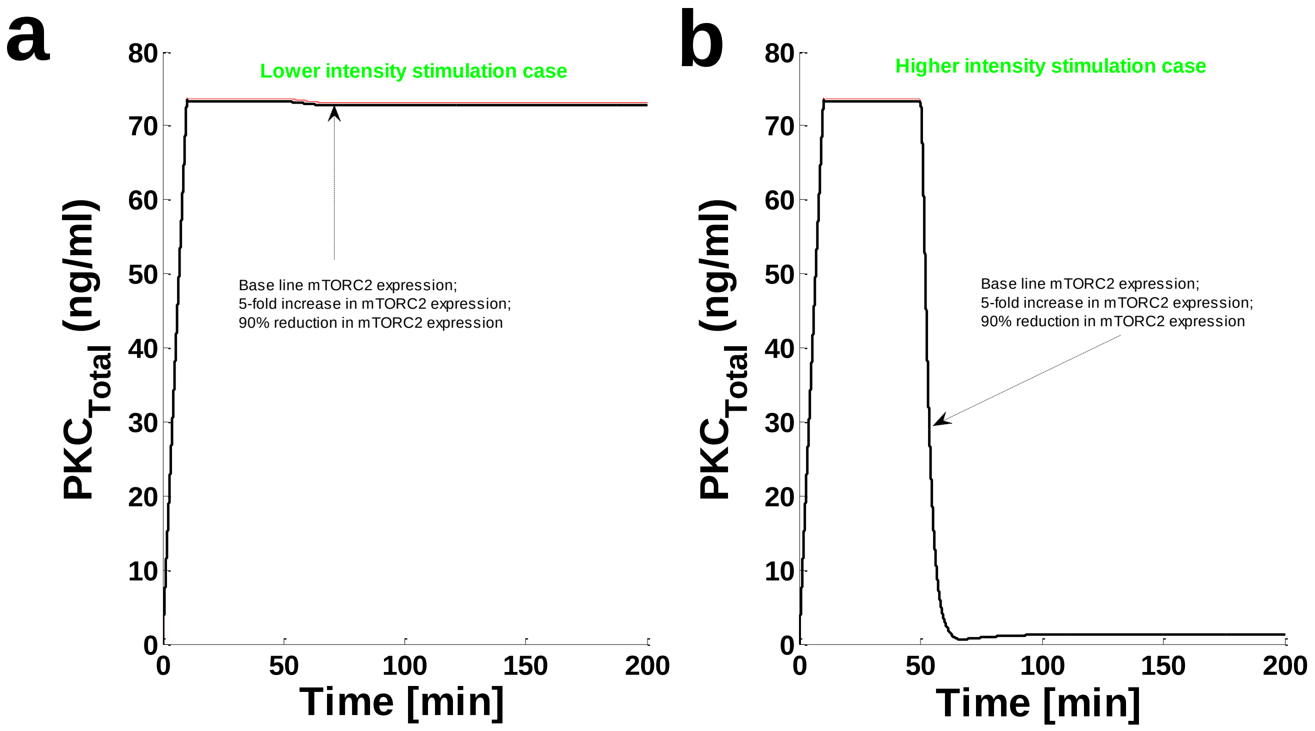

Supplement: Supplementary file 1 [file DataSheet1.PDF]
